# Non-targeted LC-MS metabolomics reveal shifts from wound-induced enzymatic browning to lignification during extended storage of fresh-cut lettuce in modified atmosphere packaging

**DOI:** 10.1016/j.crfs.2024.100959

**Published:** 2024-12-15

**Authors:** Fanny Widjaja, Priscille Steensma, Leevi Annala, Arto Klami, Saijaliisa Kangasjärvi, Mari Lehtonen, Kirsi S. Mikkonen

**Affiliations:** a Department of Food and Nutrition, University of Helsinki, P.O. Box 66, 00014, Helsinki, Finland; b Department of Computer Science, University of Helsinki, P.O. Box 68, 00014, Helsinki, Finland; c Organismal and Evolutionary Biology Research Programme, Faculty of Biological and Environmental Sciences, University of Helsinki, P.O. Box 65, 00014, Helsinki, Finland; d Department of Agricultural Sciences, Faculty of Agriculture and Forestry, University of Helsinki, P.O. Box 65, 00014, Helsinki, Finland; e Viikki Plant Science Center, University of Helsinki, P.O. Box 65, 00014, Helsinki, Finland; f Helsinki Institute of Sustainability Science (HELSUS), University of Helsinki, P.O. Box 65, 00014, Helsinki, Finland

**Keywords:** Fresh-cut vegetables, Senescence, Quality deterioration, Phenylpropanoid pathway, Hyperspectral imaging

## Abstract

Modified Atmosphere Packaging (MAP) is a conventional method used to prolong the shelf-life of fresh-cut vegetables, including lettuce. However, MAP-stored lettuce remains perishable, and its deterioration mechanism is not fully understood. Here, we utilized non-targeted LC-MS metabolomics to evaluate the effects of cutting and extended storage time on metabolite profiles of lettuce stored in MAP. Additionally, hyperspectral imaging was used to measure perceptual changes. Our findings reveal a bipartite response to wounding. In early storage, enzymatic browning was the main response to wounding, evidenced by accumulation of caffeic acid derivatives and flavonoids, substrates for polyphenol oxidases. As storage progressed, enzymatic browning was inhibited, and a shift towards lignification became apparent, evidenced by accumulation of monolignol derivatives. These findings offer new insights into the deterioration mechanism of fresh-cut lettuce occurring in MAP.

## Highlights

• Cutting and storage in MAP altered the metabolites profiles of lettuce.
• Metabolites responses were consistent with wound-induced phenylpropanoid pathway.
• Enzymatic browning progressed in early storage, evaluated by hyperspectral imaging.
• After 3 days, enzymatic browning was inhibited, and lignification became evident.
• Accumulation of lysophospholipids alongside lignification implied tissue alteration.

## Graphical abstract

**Figure:**
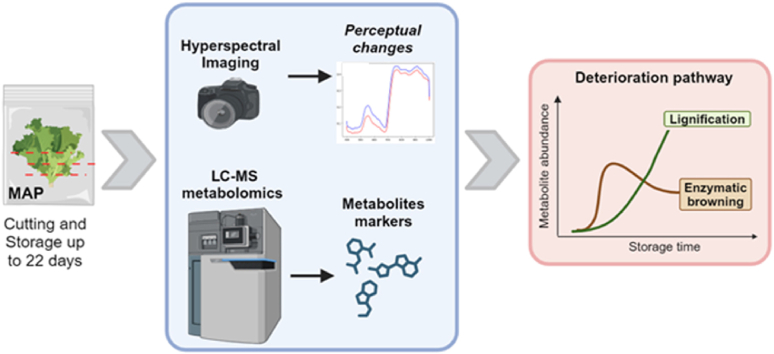

## 1 Introduction

Packaged ready-to-eat fresh-cut vegetables have recently gained popularity due to the convenience and health benefits they provide to consumers. Nevertheless, one of the big challenges in the fresh-cut vegetable market is limited shelf-life, leading to food waste and financial losses. Indeed, in 2011, the Food and Agriculture Organization of the United Nations (FAO) reported that up to 50% of produced fruits and vegetables are discarded in the distribution chain (Gustavsson et al., 2011). A significant portion of these losses result from post-harvest quality deterioration, including the development of visual blemishes, loss in flavor and texture qualities, production of off-odors, and microbiological spoilage, all of which contribute to customer rejection (Ali et al., 2018; De Corato, 2020; Peng and Simko, 2023). In the context of fresh-cut vegetables, the cutting and processing also leads to increased respiration rates and accelerated senescence (Peng and Simko, 2023; Saltveit, 2018a; Toivonen and Brummell, 2008).

One of the important packaged ready-to-eat fresh-cut vegetables is lettuce. Previous studies exploring the mechanisms of post-harvest deterioration of fresh-cut lettuce have thus far largely focused on mechanisms related to leaf discolorations such as cut-site browning, pinking, and russet spotting. Indeed, it is established that cuts to the lettuce leaves that occur during processing and storage can trigger the enzymatic oxidation of phenolic compounds leading to browning (Hunter et al., 2022; Mai and Glomb, 2013; Peng and Simko, 2023; Saltveit, 2018b). This process occurs via the phenylpropanoid pathway, which is initiated by activation of the enzyme phenylalanine ammonia lyase (PAL) leading to conversion of phenylalanine to various caffeic acid derivatives metabolites. The accumulated caffeic acid derivatives *o-*diphenols such as chlorogenic acid become substrates for polyphenol oxidases (PPO) and are converted to *o*-quinones, leading to formation of brown pigment.

Lignification is another physiological process associated with postharvest deterioration of fresh-cut vegetables (Li et al., 2024; G. L. Wang et al., 2023). Previous studies reported that the deposition of lignin in the cell wall during storage leads to undesirable textural changes, such as the hardening of carrots (Sun et al., 2024), increased firmness in Chinese flowering cabbage (L. Wang et al., 2023), and celery (Viña and Chaves, 2003). Evidence of lignification has also been reported in fresh-cut romaine, butterhead, and iceberg lettuce (García et al., 2017, 2019; Ke and Saltveit, 1988, 1989; Ripoll et al., 2019). Like enzymatic browning, lignification also occurs via the phenylpropanoid pathway, upon hydroxylation and methylation of cinnamic acid to produce key monolignols, namely *p*-coumaryl alcohol, coniferyl alcohol, and sinapyl alcohol (Dixon and Barros, 2019; Vanholme et al., 2019). These monolignols are transported to the cell wall, where oxidative enzymes such as peroxidases and laccases catalyze their polymerization into a cross-linked lignin polymer, leading to tissue hardening (Yao et al., 2021). Nowadays, the shelf-life of fresh-cut lettuce is prolonged by utilizing modified atmosphere packaging (MAP) to maintain minimum oxygen level inside the package during the storage, which reduces respiration and delays senescence, thus extends storage time (de Siqueira Oliveira et al., 2020; Guo et al., 2019; Peng and Simko, 2023). Previous studies have shown that MAP with low oxygen level can inhibit browning of cut lettuce, improving its visual quality (Chen et al., 2019; Saltveit, 2003; Tudela and Gil, 2020). Inhibition of enzymatic browning in MAP has been shown to correlate with the decrease of PPO activity which also slowed down the biosynthesis of caffeic acid derivatives (Luna et al., 2016).

Nevertheless, while MAP can successfully maintain the visual quality of lettuce by inhibiting browning development, the shelf life of MAP-stored fresh-cut lettuce is still limited due to other quality deterioration factors such as increased off-odors and decreased crispness (Cantwell and Suslow, 2002; Kim et al., 2005a, 2005b). A previous study reported that tissue deterioration in MAP-stored fresh-cut lettuce could be observed as early as 5 days after storage, depending on leaf maturity, packaging size, storage humidity, and cutting size (Peng et al., 2020). Understanding of the mechanism behind the deterioration remains incomplete and further development to prolong fresh-cut lettuce shelf-life is still of great interest, especially to meet the demand for high quality, flavorful, and fresh products with low economic and environmental cost.

Hyperspectral imaging is an advanced imaging technology that captures and processes detailed spectral data across a wide spectrum of light, from the UV to the near-infrared region, allowing detection of subtle changes that are often invisible with traditional imaging methods. Hyperspectral imaging has been increasingly used for non-destructive quality assessment of fresh fruit and vegetables, such as for predicting the firmness of apples (Lu, 2004) and peaches (Lleó et al., 2009), as well as monitoring color changes in spinach leaves (Lunadei et al., 2012) and wild rocket (Løkke et al., 2013). Recently, hyperspectral imaging was also used for monitoring the decay in fresh-cut lettuce (Simko et al., 2015).

Non-targeted LC-MS metabolomics allows for comprehensive profiling of a broad spectrum of metabolites (Cevallos-Cevallos et al., 2009; Li et al., 2021). This method combines liquid chromatography (LC) and mass spectrometry (MS) to separate, detect, and characterize metabolites across diverse chemical classes. The non-targeted approach aims to capture as many metabolites as possible, offering a holistic view of the metabolome. The resulting vast datasets, containing retention time, m/z, and intensity information, are processed using advanced bioinformatics tools to align, normalize, and annotate the detected features, often comparing them against databases for identification (Chen et al., 2022; Sumner et al., 2007). Combined with multivariate statistical analysis, this method has been used as a comparative tool to identify relevant markers in food and vegetable products (Lacalle-Bergeron et al., 2021; Utpott et al., 2022). Previously, non-targeted LC-MS metabolomics was used to evaluate metabolite variations between different lettuce varieties (Otify et al., 2023; Yang et al., 2018) as well as to reveal the biomarkers related to browning in fresh-cut lettuce stored under normal atmosphere (García et al., 2017, 2018; Liu et al., 2021).

In this study, we utilized non-targeted LC-MS metabolomics to examine how cutting and extended storage period affects the metabolite composition of lettuce packed in modified atmosphere with limited oxygen. The changes in metabolite profiles of cut (detached shredded leaves) and non-cut (detached intact leaves) lettuce stored in MAP from early (3 h) to later stages of storage (22 days) were recorded and compared, and the key metabolites involved in quality deterioration process related to cutting and storage in MAP were identified. Additionally, changes in the metabolite content were linked to variations in the lettuce leaves quality attributes, such as degree of browning and leaves water content, as evaluated with hyperspectral imaging and spectrophotometric analysis.

The aim of this study was to elucidate the key biochemical pathways underlying post-harvest deterioration of fresh-cut lettuce upon cutting and storage in MAP. This insight could provide essential information for the future development of novel processing and packaging techniques to further prolong the shelf life of fresh-cut vegetables.

## 2 Methods

### 2.1 Chemicals and reagents

HPLC grade methanol and HPLC grade isopropanol used for sample extraction, and LC-MS grade acetonitrile used as HPLC mobile phase were purchased from Honeywell Riedel-de Haën (Germany). Acetic acid (glacial ≥99%) used for sample extraction was purchased from Fisher Scientific (United Kingdom) and LC-MS grade formic acid (>99%) used as HPLC mobile phase was purchased from VWR International (United Kingdom). Chlorogenic acid (analytical standard) used as reference standard for metabolites identification was purchased from Sigma-Aldrich (St. Louis, MO, USA). Water was purified by a Milli-Q Plus system (Millipore Corporation Bedford, MA, USA). All chemicals were used as received.

### 2.2 Plant materials

Fresh lettuces (*Lactuca sativa,* variety Crispiano, Eazyleaf®, Enza zaden, The Netherlands) were purchased from Robbes Lilla Trädgård Ab (Lindkoski, Finland) for two experimental replicates (Experiment 1, January 2023 and Experiment 2, August 2023). The lettuces were grown hydroponically for 2–3 weeks in a vertical farming system whereafter replanted in a greenhouse maintained at 20 °C and 75% relative humidity under 18 h photoperiod and 6 h darkness. The lettuces were harvested at the age of 40 days. They were delivered to the laboratory within 24 h after collection from the greenhouse (roots and soil still attached and plants wrapped in protective plastic) by cooled transportation (approximately 10 °C) and processed within 4 h of delivery.

#### 2.2.1 Lettuce processing, packaging, and storage

Upon delivery, each of the lettuce leaves were carefully removed from the head. Two lettuce leaves located in the middle part of the head were selected, to ensure they were of similar age and maturity. Two types of samples were prepared from the selected leaves, packaged cut lettuce leaves (cut samples) and packaged non-cut lettuce leaves (non-cut samples).

To prepare cut samples, the entire leaves were cut perpendicularly to the midrib into 1-cm strips. To prepare non-cut samples, the remaining stem tissue was cut (about 1 cm) from the detached leaves and they were used without further processing. The two leaves (cut or non-cut) from each lettuce head weighed between 10 and 20 g. The leaves were not washed to ensure that none of the relevant metabolites were removed or diluted. The cut and non-cut lettuces were thereafter packed and sealed under modified atmosphere conditions with a starting gas composition of 5% O_2_, 10% CO_2_ and 85% N_2_, using a Max F-46 packaging machine (Vacuum Boss, Friedrichsdorf, Germany). MAP gas composition was chosen following recommendation from (Saltveit, 2003). The packaging film was received from FreshServant Oy (Seinäjoki, Finland), a local distributor of fresh-cut vegetable products in Finland. The film material was a complex of biaxially oriented polypropylene (20 μm) and anti-fog polypropylene (25 μm), with oxygen and carbon dioxide permeability less than 848 and 3389 cm^3^/m^2^/24h, respectively. The film was cut into 25 cm × 25 cm pieces and sealed, to create a bag with a volume of approximately 265 cm^3^ for packaging of the lettuce.

The packaged lettuce leaves were stored in a climatic chamber (Aralab, Portugal) under darkness at 7 °C, and 50% relative humidity for various storage times (T_x_) after processing and packaging. When the desired storage time had been reached, 3 biological replicates per each sample type (cut and non-cut) were withdrawn. At each collection time, headspace gas composition (O_2_ and CO_2_) inside the packages was determined using a CheckMate 9900 O_2_/CO_2_ gas analyzer (PBI-Dansensor, Ringsted, Denmark) and the weight of the lettuce leaves after storage were recorded. Digital images of the lettuce leaves were taken to visualize quality deterioration such as browning and/or discoloration. The leaves were then frozen immediately by dipping them into liquid nitrogen. Additionally, cut and non-cut fresh (T_0_) samples were also prepared. For these samples (T_0_), the leaves were not packed nor put in storage, but were frozen immediately after processing. All of the frozen lettuce leaves were stored in −60 °C for a maximum of 3 months prior to further processing.

The frozen lettuce leaves were homogenized into a fine powder using a Grindomax GM300 (Retsch, Haan, Germany) under cryogenic milling and ambient light conditions. The frozen powders were stored in polypropylene tubes (Sarstedt, Germany) in darkness at −60 °C for spectrophotometric and non-targeted LC-MS analysis, all of which were performed within 12 months of sample processing and packaging.

### 2.3 Spectrophotometric analysis

To evaluate degree of browning of the lettuce, spectrophotometric analysis was performed following the method described in previous study (Mai and Glomb, 2013). In brief, 1 g of ground sample was extracted with 1 mL of methanol-water mixture (4:1, v/v) under ultrasonication for 30 min. The slurry was then vortexed and centrifuged at 10000*g* for 15 min. Supernatant was collected and filtered with a 0.22 μm PVDF syringe filter (Pall Lab, USA). 200 μL of the supernatant from each sample was transferred to a transparent 96-wells plate (Thermo Fisher Scientific, USA). The absorbance values at 420 nm were determined using a Varioskan LUX plate reader (Thermo Fisher Scientific, USA).

### 2.4 Hyperspectral imaging

Hyperspectral imaging was performed to further confirm the degree of browning of the lettuce as well as evaluation of their water content. For this analysis, additional cut and non-cut lettuce samples from Experiment 2 corresponding to one biological replicate per storage timepoint were prepared.

Hyperspectral images were taken using a portable hyperspectral camera Specim IQ (Specim, Spectral Imaging Ltd., Oulu, Finland) with a spectral range from 400 to 1000 nm. The camera was placed on a tripod approximately 35 cm above the sample. Two Effilux EFFI-Flex-HSI extended spectrum lamps (Effilux, France) were used during imaging, and they were placed at a 45° angle, 30 cm from each side of the sample. Images were taken in custom imaging mode, following instructions in the Specim IQ User Manual. Two additional white reference hyperspectral images were taken, one before the first sample images and another after the last sample images, to account for spatial and temporal changes in light conditions. The integration time was locked at 51 ms to maximize data quality while avoiding over- and undersaturation of the hyperspectral image.

The hyperspectral images were processed using Python programming language (v. 3.10.12), and python packages xarray (v. 2023.9.0), NumPy (v. 1.26.0), OpenCV (v. 4.8.1) SciPy (v. 1.11.3). First, foreground extraction was carried out to distinguish the sample image from the background. This was performed using the ratio of the reflectance at 543 nm, where there is the most distinct difference between the sample image and the background, and the reflectance at 470 nm, where there is the least difference between the sample image and the background. The threshold of this ratio was fixed at 1 and the approximate map of the sample foreground was obtained. After foreground extraction, shadow removal was performed (Winkens and Paulus, 2019). The hyperspectral images were split into infrared and visible light domains, and the pixels that are dim in both domains were considered as shadow. Using this method, approximately 25% of the total pixels from each hyperspectral image were removed from the analysis.

The processed hyperspectral images were then used for evaluation of degree of browning of cut and non-cut lettuce leaves. Degree of browning was evaluated using Browning Reflectance Index (BRI), calculated as (1/R_550_ - 1/R_700_)/R_893_, where R_550_, R_700_, and R_893_ are the sample reflectance at 550 nm, 700 nm, and 893 nm (Merzlyak et al., 2003). Additionally, the hyperspectral images were also used for evaluation of the water content of the samples, by calculating Water Index (WI), calculated as R_970_/R_900_ (Ceccato et al., 2002). To correlate the results, the leaves were dried for 24 h at 80 °C and the water content in the leaves tissue were directly calculated based on the difference between the fresh weight and the dried weight of the samples (Agüero et al., 2008).

### 2.5 Non-targeted LC-MS metabolomics

#### 2.5.1 Sample extraction

To efficiently extract as many metabolites as possible from the lettuce samples, two different sample extraction methods were used: non-acidic and acidic. Non-acidic extraction was performed according to the method described in previous study (Llorach et al., 2008), with minor modifications. Briefly, 300 mg of ground lettuce samples were extracted with 300 μL of a methanol-water mixture (4:1, v/v) at room temperature by ultrasonication for 30 min. The slurry was vortexed and centrifuged at 10000*g* for 15 min at 4 °C. Supernatant was filtered with 0.22 μm PVDF syringe filter for LC-MS analysis. For acidic extraction, the same procedure was performed but using methanol-isopropanol-acetic acid mixture (20:79:1, v/v) under ice (2–8 °C). Extracted samples were stored at 4 °C for a maximum of 3 days prior to LC-MS analysis.

#### 2.5.2 LC-MS analysis

LC-MS analysis was performed using Waters Acquity UPLC coupled with Synapt G2-Si Quadrupole Time-of-Flight mass spectrometer (Waters, Milford, MA, USA). Chromatography separation was achieved using Waters Acquity Premier BEH C18 column (2.1 × 100 mm, 1.7 μm) at 40 °C and utilizing a combination of water with 0.1% formic acid (A) and acetonitrile with 0.1% formic acid (B) as mobile phase with flow rate of 0.2 mL/min 5 μL of lettuce extract was injected into the column, run with a linear gradient from 10% to 90% B over 15 min.

MS spectra were acquired using negative mode electrospray ionization with m/z range set for 50–1200. To ensure accuracy, a reference lock-mass calibration using leucine-enkephalin was performed. Lock mass data were acquired at 10 s interval and averaged every 3 spectra. Initial MS/MS analysis were performed using MS^E^ mode, after which targeted MS/MS were performed to increase the confidence in compound identification. The following instrument settings were used for MS analysis: capillary voltage 2.5 kV, sampling cone 25 V, source offset 80 V, source temperature 120 °C, desolvation temperature 500 °C, cone gas flow 50 L/h, desolvation gas flow 1000 L/h, nebulizer gas flow 6.5 bar, trap collision energy 4 eV, trap gas flow 2 mL/min, and scan time of 0.2 s. For MS^E^ and MS/MS analysis, the same settings were used except that for MS/MS analysis the trap collision energy was ramped from 10 to 40 eV to obtain sufficient fragment ions.

Data analysis including peak alignment, peak picking, ion deconvolution, and ion intensity normalization was performed by utilizing Progenesis QI 3.0.3 software (Nonlinear Dynamics, Newcastle, UK). The lettuce samples were grouped based on processing type (cut vs. non-cut) and storage time (T_x_). One-way ANOVA with *p* ≤ 0.05 was applied and compound entities with variation in normalized intensity larger than 30% within the sample group were excluded. Partial least square discriminant analysis (PLS-DA) was performed to discover the most significant metabolite features differentiating the lettuce samples (EZinfo For Progenesis QI 2.3 software, Umetrics, Sweden). Compounds with variable of importance in projection score (VIP score) greater or equal to 1 were considered to be the most significant metabolite features. These metabolites were tentatively identified based on their accurate mass and molecular formula (mass error ≤ 10 ppm, isotope similarity ≥90%), as well as comparison of their MS/MS fragments with online databases (e.g. ChemSpider, PubChem, Lipidmap, FooDB) and other literature data (Abu-Reidah et al., 2013; Kiyota et al., 2012; Otify et al., 2023; Yang et al., 2018). The identified metabolites were then classified into different compound groups based on their structural characteristics.

LC-MS data analysis was performed separately for non-acidic and acidic extracts. The identified metabolites from both extraction methods were compared and combined. For overlapping metabolites, the data were selected based on the method that yielded the highest ion intensities. Due to the lack of authentic standards, a semi-quantitative approach using LC-MS raw ion abundance was used to evaluate the changes in metabolite abundances in the lettuce samples. To evaluate the effect of cutting, metabolite abundances in cut samples were compared to those in non-cut samples. To evaluate the effect of storage time, metabolite abundances along storage time (T_x_) were compared to those in fresh (T_0_) samples. For each timepoint, the average relative changes from three biological replicates were calculated.

## 3 Results

### 3.1 Headspace gas composition in the lettuce packages during storage

**Fig. 1:**
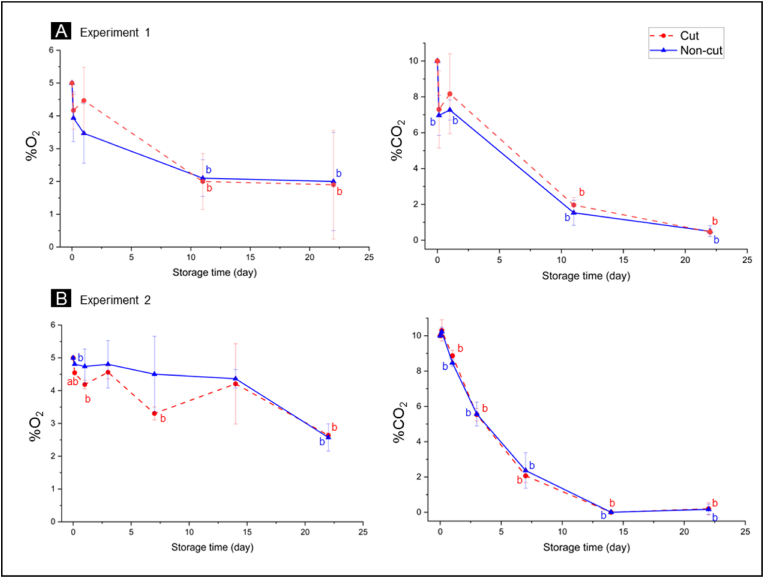
Headspace gas composition (O_2_ and CO_2_) inside the packages containing cut and non-cut lettuce during storage in MAP (Experiment 1, top and Experiment 2, bottom). The data represent mean ± SD across three biological replicates per storage time. Statistical analysis was performed using a 2-tailed Student's t-test, where *a* indicates p ≤ 0.05 when compared to non-cut samples and *b* indicates p ≤ 0.05 when compared to T_0_ samples.

Respiration is a key process that drives the post-harvest deterioration of fresh-cut lettuce, affecting its metabolic processes. In this study, we used MAP with starting gas composition of 5% O_2_ and 10% CO_2_ to reduce the respiration rate and delay deterioration. We measured the headspace gas composition (O_2_ and CO_2_ content) inside the packages at each storage time (Fig. 1), to ensure consistent and controlled storage across biological replicates. Additionally, it is important to monitor the variations in MAP gas composition over the storage period, as these variations can significantly influence the metabolite profiles of lettuce and contribute to physiological differences between samples.

We observed minimal variation (<2%) in both O_2_ and CO_2_ gas composition across biological replicates (n = 3). Furthermore, we observed similar trends across storage period for both Experiment 1 and Experiment 2. Throughout the 22-day storage period, no significant differences in headspace gas composition were detected between packages containing cut and non-cut lettuce. However, changes in headspace composition were noted as storage progressed. During the 22-day storage period, low O_2_ level was maintained, but decreasing from 5% to approximately 2–3%. In contrast, CO_2_ level was decreasing more rapidly and fell below detection limits (<0.1%) after 14 days of storage. The decrease in CO_2_ level was likely due to the intrinsic permeability of the packaging film, allowing CO_2_ to quickly diffuse out of the package to prevent fermentation and CO_2_ injury. This suggests that headspace composition is unlikely to contribute to the physiological differences between cut and non-cut lettuce. However, it might contribute to the changes occurring in the samples as the storage progresses.

### 3.2 Visual changes in cut and non-cut lettuce during storage

**Fig. 2:**
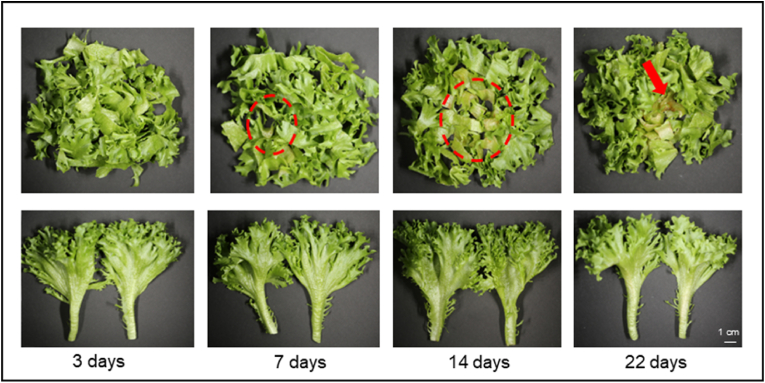
Digital images of cut and non-cut lettuce (Experiment 2) after 3, 7, 14, and 22 days of storage in MAP. Apparent browning is indicated with a circle and tissue softening with a red arrow.

Visual inspection of cut and non-cut lettuce (Fig. 2) suggested only minimal browning in cut lettuce even after 7 days of storage. Further development of browning on the cut edges of the midribs was apparent until 14 days but did not appear to progress after that. Additionally, tissue softening was apparent in cut lettuce after 22 days. For non-cut lettuce, browning occurred only at the edge of the midrib, where the leaves were detached. No other visual signs of deterioration were observed during 22 days of storage.

To confirm visual observation of browning, the absorbance of sample extracts at 420 nm was recorded spectrophotometrically. Additionally, degree of browning was also evaluated from the hyperspectral images of the lettuce samples by calculating the Browning Reflectance Index (BRI).

**Fig. 3:**
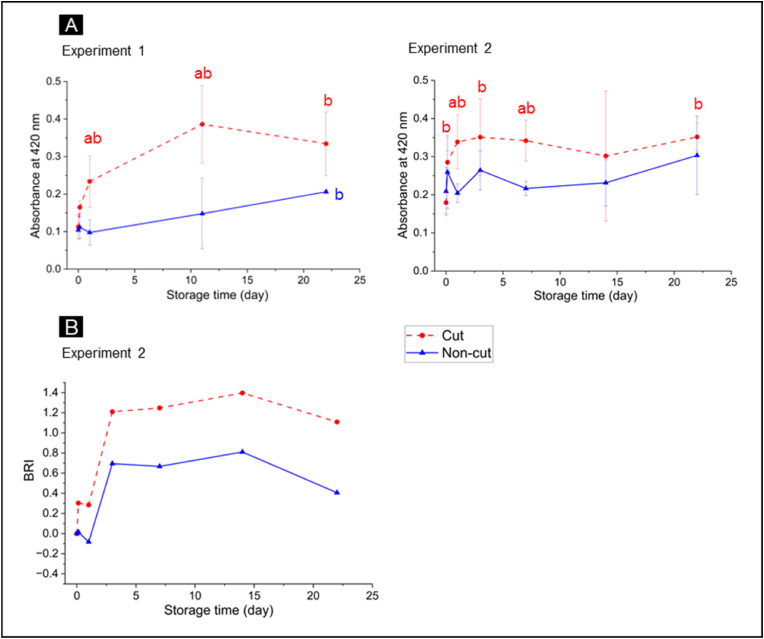
Degree of browning in cut and non-cut lettuce during storage in MAP, assessed by spectrophotometric analysis (A) and hyperspectral imaging (B). Degree of browning was measured based on absorbance at 420 nm and Browning Reflectance Index (BRI), respectively. For spectrophotometric analysis, the data represent mean ± SD across three biological replicates per storage time. For hyperspectral imaging analysis, the data represent one biological replicate per storage time. Statistical analysis was performed using a 2-tailed Student's t-test, where *a* indicates p ≤ 0.05 when compared to non-cut samples and *b* indicates p ≤ 0.05 when compared to T_0_ samples.

Both spectrophotometric results and hyperspectral imaging analysis showed a significant difference in the degree of browning between cut and non-cut lettuce (Fig. 3). Higher degree of browning was observed in cut lettuce compared to non-cut lettuce, which correlates well with the visual observation, particularly after 7 days of storage (Fig. 2). Along storage time, a significant increase in the degree of browning was apparent in cut lettuce already after 1–3 days of storage (Fig. 3) after which the progression of browning plateaued and the degree of browning remained relatively constant until the end of the storage period. These results suggest that under our experimental conditions, cutting significantly accelerates browning, but the progression of browning was inhibited after 3 days of MAP storage.

Nevertheless, while progression of browning within the first 3 days of storage was detected by spectrophotometric and hyperspectral imaging analysis, it did not result in visible changes to the lettuce (Fig. 2). This may be due to the higher sensitivity of these methods, which detect browning earlier than can be seen through visual observation.

### 3.3 Water loss during storage

**Fig. 4:**
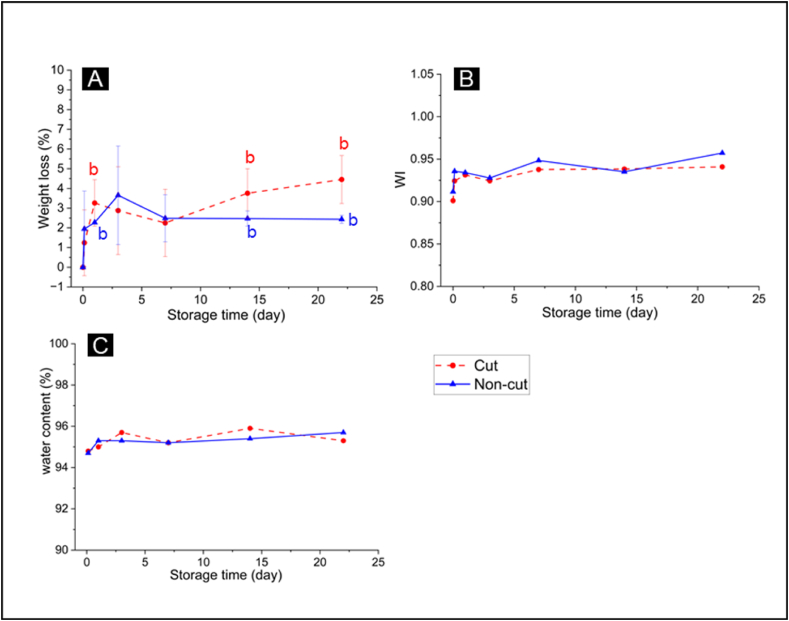
Water loss in cut and non-cut lettuce during storage in MAP, assessed by measurement of weight loss (A) and calculation of Water Index (WI) from hyperspectral imaging analysis (B). The results were further confirmed with measurement of the lettuce water content using dried samples (C). For measurement of weight loss, the data represent mean ± SD across three biological replicates per storage time. For hyperspectral imaging and water content analysis, the data represent one biological replicate per storage time. Statistical analysis was performed using a 2-tailed Student's t-test, where *a* indicates p ≤ 0.05 when compared to non-cut samples and *b* indicates p ≤ 0.05 when compared to T_0_ samples.

In addition to browning, water loss is another indication of quality deterioration in lettuce. To monitor water loss during storage, the weight change of the lettuce before and after storage were recorded (Fig. 4A). We observed minimal weight changes to both cut and non-cut lettuce throughout the storage. At 22 days of storage, the cut and non-cut lettuce had an average of only 4% and 2% weight loss, respectively.

Water loss was also evaluated from the hyperspectral images of the lettuce by calculating the Water Index (WI) (Fig. 4B). The results were confirmed with direct measurement of the lettuce water content, based on the difference in the dry weight and fresh weight of the samples (Fig. 4C). Both measurements suggested that there was no significant difference in the water content of the cut and non-cut lettuce even after 22 days of storage. This result suggests that under our experimental conditions, both cutting and MAP storage minimally impact the water content of the samples.

### 3.4 Changes in the metabolite profiles of lettuce due to cutting and storage

To further understand the quality deterioration mechanism, we evaluated changes in the metabolite profiles of lettuce due to cutting and storage in MAP by utilizing non-targeted LC-MS analysis. A large number of metabolites were detected in the lettuce extracts. In Experiment 1, 8827 metabolites were detected in non-acidic extracts and 6133 in acidic extracts, and in Experiment 2, 8633 and 5404 metabolites, respectively.

**Fig. 5:**
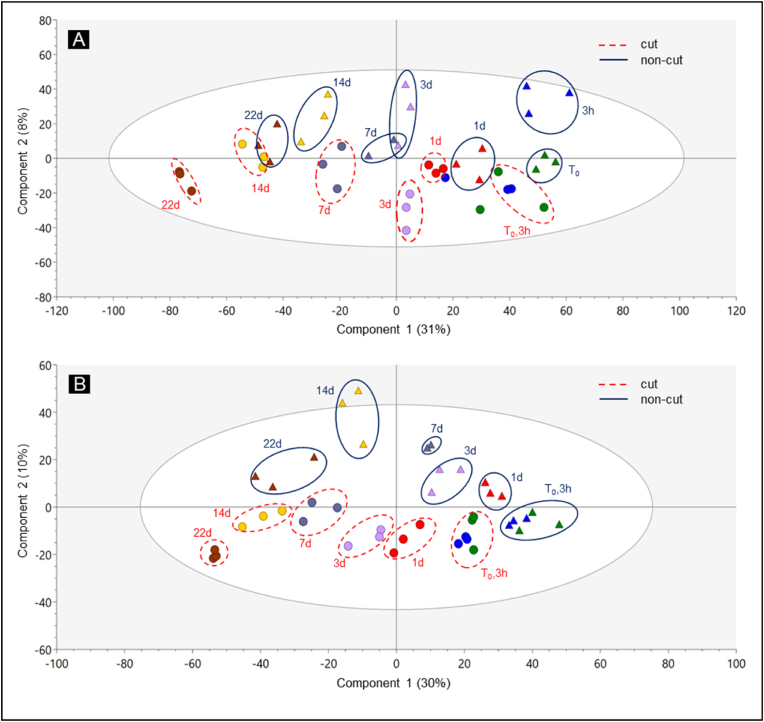
PLS-DA score plots (Experiment 2 samples; A: non-acidic extracts, B: acidic extracts) describing the differences in metabolite profiles of lettuce due to cutting and storage in MAP. Cut and non-cut lettuce are denoted as (•) and (▲), different colors refer to different storage times (n = 3).

To evaluate the effect of cutting and storage time on metabolite profiles of lettuce, partial least square discriminant analysis (PLS-DA) was performed. The model explained 39–49% of the total variance for non-acidic extracts and 40–45% for acidic extracts. Subsequently, a PLS-DA score plot was used to visualize the correlations between the sample groups (Experiment 2, Fig. 5; Experiment 1, Supplementary Data Fig. S1). Experiment 2 is presented here since it included more sampling time points, allowing closer investigation on the effects of storage time. The effect of storage time was apparent in Component 1, which accounted for 30–31% of the sample differences, whereas the effect of cutting was apparent in Component 2, which accounted for 8–10% of the sample differences (Fig. 5). This result suggests that storage time has a more significant effect on metabolite profiles than cutting.

### 3.5 Key metabolites explaining the observed differences due to cutting and storage

We identified thirty key metabolites differentiating the lettuce samples upon cutting and storage in MAP (Table 1). MS/MS fragments (Supplementary Data, Table S1) were also used to aid metabolites identification. The identified metabolites were categorized into different compound groups, namely lysophospholipids, oxylipins, jasmonates, amino acids, vitamin B5, and phenolic metabolites, which were further sub-grouped as coumaric acid derivatives, caffeic acid derivatives, flavonoids, and monolignol derivatives.

**Table 1:** Key metabolites differentiating lettuce upon cutting and storage in MAP.

#	Retention time (min.)	Observed m/z [M-H]^-^	Molecular formula	Calculated m/z [M-H]^-^	Mass error (ppm)	Tentative ID
*Lysophospholipids*						
1	13.2	474.261	C_23_H_42_NO_7_P	474.263	−3.0	PE(18:3/0:0)(1)^a^^b^
2	13.5	474.261	C_23_H_42_NO_7_P	474.263	−3.2	PE(18:3/0:0)(2)^a^^b^
3	14.2	476.277	C_23_H_44_NO_7_P	476.278	−2.7	PE(18:2/0:0)(1)^a^^b^
4	14.5	476.277	C_23_H_44_NO_7_P	476.278	−3.1	PE(18:2/0:0)(2)^a^^b^
5	15.3	452.277	C_21_H_44_NO_7_P	452.278	−3.7	PE(16:0/0:0)^b^
*Oxylipins: C14 and C16 oxylipins*						
6	7.4	273.169	C_14_H_26_O_5_	273.171	−6.0	Hydroxytetradecanedioic acid^c^
7	8.5	301.200	C_16_H_30_O_5_	301.202	−6.2	Hydroxyhexadecanedioic acid^c^
*Oxylipins: C18 oxylipins*						
8	9.3	327.216	C_18_H_32_O_5_	327.218	−3.6	Trihydroxyoctadecadienoic acid^c^
9	9.8	329.232	C_18_H_34_O_5_	329.233	−3.5	Trihydroxyoctadecenoic acid^c^
*Jasmonates*						
10	8.1	227.127	C_12_H_20_O_4_	227.129	−7.3	12-Hydroxy-9,10-dihydrojasmonic acid^c^
11	11.0	322.201	C_18_H_29_NO_4_	322.202	−3.0	Jasmonoyl isoleucine^c^
*Amino acids and Vitamin B5*						
12	2.1	164.070	C_9_H_11_NO_2_	164.072	−7.3	Phenylalanine^c^
13	3.2	203.082	C_11_H_12_N_2_O_2_	203.083	−4.7	Tryptophan^c^
14	2.1	218.102	C_9_H_17_NO_5_	218.103	−6.9	Vitamin B5^d^
*Phenolic metabolites: coumaric acid derivatives*						
15	4.2	295.044	C_13_H_12_O_8_	295.046	−6.4	Coumaroyltartaric acid^d^
16	5.1	337.092	C_16_H_18_O_8_	337.093	−3.2	Coumaroylquinic acid (1)^a^^c^
17	5.7	337.092	C_16_H_18_O_8_	337.093	−3.0	Coumaroylquinic acid (2)^a^^c^
*Phenolic metabolites: caffeic acid derivatives*						
18	2.9	311.039	C_13_H_12_O_9_	311.041	−6.6	Caffeoyltartaric acid^e^
19	3.9	353.087	C_16_H_18_O_9_	353.088	−3.4	Caffeoylquinic acid (1)^a^^e^
20	5.0	353.087	C_16_H_18_O_9_	353.088	−3.4	Caffeoylquinic acid (2)^a^^e^
21	6.1	473.071	C_22_H_18_O_12_	473.073	−2.9	Dicaffeoyltartaric acid^e^
22	6.7	515.119	C_25_H_24_O_12_	515.120	−1.4	Dicaffeoylquinic acid^e^
*Phenolic metabolites: flavonoids*						
23	6.2	477.066	C_21_H_18_O_13_	477.068	−2.7	Quercetin-3′-glucuronide^e^
24	6.4	505.098	C_23_H_22_O_13_	505.099	−1.4	Quercetin-3'-(6″-acetylglucoside)^e^
*Phenolic metabolites: monolignol derivatives*						
25	3.8	385.117	C_17_H_22_O_10_	385.114	8.5	Sinapoyl glucoside^d^
26	4.7	355.102	C_16_H_20_O_9_	355.104	−2.4	Ferulic acid-O-glucoside^d^
27	5.0	209.080	C_11_H_14_O_4_	209.082	−7.5	Sinapyl alcohol^d^
28	5.1	207.065	C_11_H_12_O_4_	207.066	−7.3	Sinapaldehyde^c^
29	6.8	341.126	C_16_H_22_O_8_	341.124	3.9	Coniferin^c^
30	7.1	417.156	C_22_H_26_O_8_	417.156	2.2	Syringaresinol^d^

a Multiple isomers were detected.

b Compounds detected only in acidic extracts.

c Compounds detected in both non-acidic and acidic extracts, but non-acidic extracts yielded higher abundance.

d Compounds detected only in non-acidic extracts.

e Compounds detected in both non-acidic and acidic extracts, but acidic extracts yielded higher abundance.

Five lysophospholipids belonging to the monoacyl glycerophosphoethanolamine class were identified. This phospholipid class produces a characteristic MS/MS fragment ion of m/z 152.996 (calculated exact mass), indicating the presence of a phosphoglyceryl moiety. All the detected lysophospholipids generated such fragment, confirming their subclassification. In addition, Compounds 1 and 2 produced characteristic fragment ions m/z 277.218, indicating the presence of an octadecatrienoyl (C18:3) moiety (Supplementary Data, Fig. S4). Compounds 3 and 4 produced fragment ions m/z 279.229 and compound 5 produced fragment ions m/z 255.231, indicating octadecadienoyl (C18:2) and hexadecanoyl (C16:0) moieties, respectively (Supplementary Data, Figs. S5–S6).

Two oxylipins (Compound 6 and 7) were identified as C14 and C16 hydroxylipins containing both carboxyl and hydroxyl groups. Both compounds underwent a neutral loss of 100 Da, which corresponds to loss of a carboxyl group (−44 Da) followed by loss of C_4_H_8_ (−56 Da). The generated fragment ion then underwent a loss of water, indicating the presence of a hydroxyl group (Supplementary Data, Figs. S7–S8). Two other oxylipins, Compounds 8 and 9, were identified as C18 oxylipins having octadecadienoyl (C18:2) and octadecenoyl (C18:1) units and containing three hydroxyl groups, respectively. Both compounds produced the same fragment ion m/z 211.130 (C_12_H_19_O_3_^−^), upon neutral loss of hexenal (C_6_H_10_O) or hexanal (C_6_H_12_O) followed by loss of water (Supplementary Data, Figs. S9–S10). This fragmentation pattern indicated the presence of three hydroxyl groups.

Two jasmonate derivatives were identified: 12-hydroxy-9,10-dihydrojasmonic acid (Compound 10) and jasmonoyl isoleucine (Compound 11). Compound 10 underwent a loss of water producing fragment ion m/z 209.117, corresponding to deprotonated jasmonic acid ion. Compound 11 fragmented underwent a loss of jasmonoyl acid producing fragment ion m/z 130.087 which corresponds to deprotonated isoleucine ion (Supplementary Data, Fig. S11).

The majority of the identified key metabolites were phenolic compounds (16 out of 30), which can be sub-grouped as coumaric acid derivatives, caffeic acid derivatives, flavonoids, and monolignol derivatives. Coumaric acid and caffeic acid derivatives produced characteristics fragments ions m/z 163.040 and m/z 179.034 (calculated exact masses), corresponding to coumaroyl and caffeoyl moieties, respectively. Other fragment ions such as m/z 149.009 and m/z 191.056 were indicative of either tartaroyl or quinoyl moieties. Compounds 15–22 generated similar fragments (Supplementary Data, Table S1, and Figs. S12–S17), confirming their identification. Additionally, the identity of Compound 19 was further confirmed as 3-O-caffeoylquinic acid (chlorogenic acid) based on comparison with the reference standard.

Two flavonoids were identified as *O*-glycosides, and further as quercetin derivatives (Compounds 23 and 24). Both compounds produced characteristic fragment ion m/z 301.037, corresponding to deprotonated quercetin ion due to loss of glucuronyl and acetylglucosyl moieties, respectively (Supplementary Data, Figs. S18–S19).

**Fig. 6:**
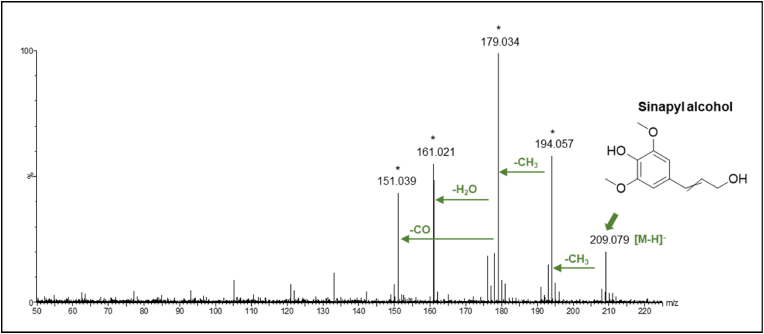
MS/MS spectrum of Compound 27 (Table 1) identified as sinapyl alcohol (parent ion [M-H]^-^, m/z 209.079). Major fragment ions and neutral losses were indicated with ∗.

Metabolites belonging to both S- (sinapyl alcohol) and G- (coniferyl alcohol) monolignol derivatives were also identified. The fragmentation pattern of these compounds followed the pattern of methoxylated cinnamic acid, that is by losses of methyl and/or carbonyl groups (Supplementary Data, Figs. S20–S23). For example, Compound 27, identified as sinapyl alcohol, produced fragment ion m/z 179.034 after consecutive losses of two methyl groups. Further fragmentation owing to the loss of carbonyl group or water were also observed (Fig. 6). A similar fragmentation pattern was also detected for Compound 28, identified as sinapaldehyde (Supplementary Data, Fig. S22).

**Fig. 7:**
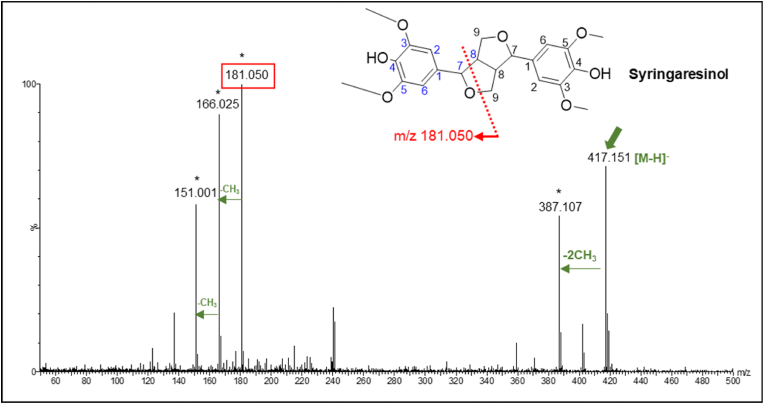
MS/MS spectrum of Compound 30 (Table 1) identified as syringaresinol (parent ion [M-H]^-^, m/z 417.151). Major fragment ions and neutral losses were indicated with ∗.

Additionally, a lignan, identified as syringaresinol (Compound 30) was detected as one of the key metabolites. This compound produced a characteristic fragment ion m/z 181.050, indicating the presence of an 8-8 resinol link between two S-monolignol units (Fig. 7). This ion fragmented further by loss of either one or two methyl groups from the S-monolignol unit producing fragment ions m/z 166.025 and m/z 151.001.

### 3.6 Effect of cutting and storage time on metabolite abundances

**Fig. 8:**
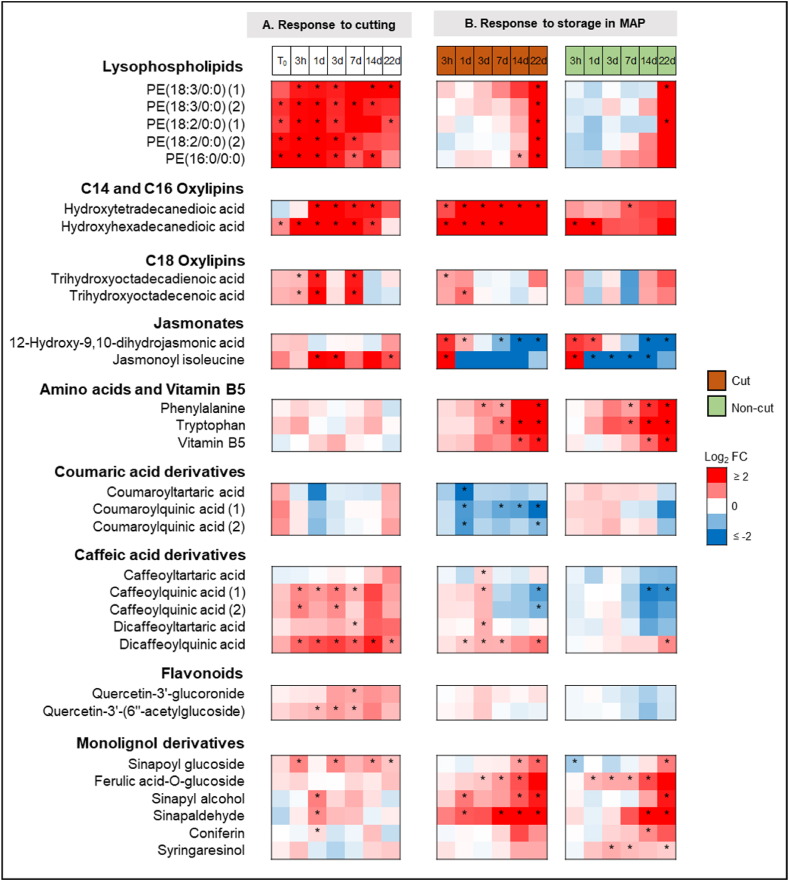
Metabolite response to cutting (A) and storage in MAP (B) from Experiment 2 samples. Heatmaps represent the log_2_ fold change (log_2_ FC) of metabolite abundances between A) cut vs. non-cut lettuce at each storage time (T_x_) and B) stored (T_x_) vs. fresh (T_0_) for both cut and non-cut lettuce. The data represent the mean of three biological replicates per storage time. Statistical analysis was performed using a 2-tailed Student's t-test, where *∗* indicates p ≤ 0.05.

**Fig. 9:**
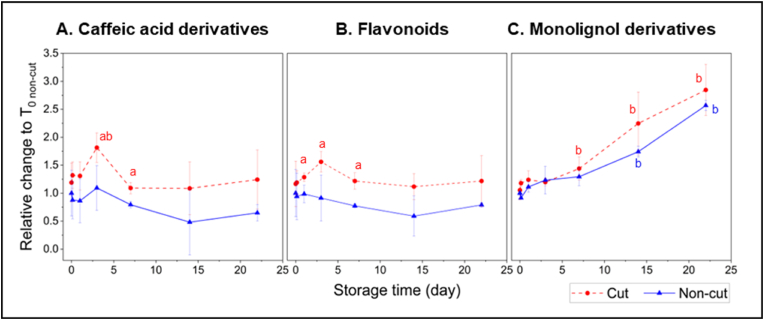
Accumulation of caffeic acid derivatives (A), flavonoids (B), and monolignol derivatives (C) in cut and non-cut lettuce stored in MAP (Experiment 2). Relative changes are represented in comparison to fresh non-cut lettuce samples (T_0 non-cut_). The data represent the mean ± SD of three biological replicates per storage time. Statistical analysis was performed using a 2-tailed Student's t-test, where *a* indicates p ≤ 0.05 when compared to non-cut samples and *b* indicates p ≤ 0.05 when compared to T_0_ samples.

To evaluate the effect of cutting, metabolite abundances in the cut lettuce were compared relative to those in the non-cut lettuce at different storage times (Experiment 2, Fig. 8A; Experiment 1, Supplementary Data Fig. S2A). To evaluate the effect of storage time, metabolite abundances in both cut and non-cut lettuce along storage time (T_x_) were compared relative to those in fresh (T_0_) lettuce samples (Experiment 2, Fig. 8B; Experiment 1, Supplementary Data Fig. S2B). Additionally, the abundances of all caffeic acid derivatives, all flavonoids, and all monolignol derivatives metabolites were compared relative to those in fresh (T_0_) non-cut lettuce samples (Experiment 2, Fig. 9; Experiment 1, Supplementary Data Fig. S3) to assess the overall changes between these compound groups.

Cutting resulted in a significant increase in metabolite abundances for all lysophospholipids, all oxylipins, jasmonoyl isoleucine, and most of the phenolic metabolites (Fig. 8A). For all lysophospholipids, C14 and C16 oxylipins, significant accumulation due to cutting was observed throughout 22 days storage period. For C18 oxylipins and jasmonoyl isoleucine, the greatest accumulation was observed in cut lettuce stored for 1 day. Cutting also resulted in significant accumulation of caffeic acid derivatives, flavonoids, and monolignol derivatives, but the greatest accumulation was observed for caffeic acid derivatives, particularly in cut lettuce stored for 3 days (Fig. 9).

Along storage time, significant changes in metabolite abundances were observed in both cut and non-cut lettuce (Fig. 8B). Notably, an increase in abundance throughout storage was observed for all lysophospholipids, C14 and C16 oxylipins, amino acids and Vitamin B5. In contrast, a decrease was observed for all coumaric acid derivatives. For C18 oxylipins and jasmonates, metabolite abundances increased during the first 3 h or 1 day of storage, after which decreasing trends were observed.

Different abundance trends over storage time were observed for caffeic acid derivatives, flavonoids, and monolignol derivatives (Fig. 9). For caffeic acid derivatives and flavonoids, increasing metabolite abundances were observed in cut lettuce stored within early storage times, with maximum accumulation in samples stored for 3 days. After that, metabolite abundances decreased and remained relatively constant until the end of the 22-day storage period. A similar trend was observed in non-cut lettuce samples, although the changes were not statistically significant. For monolignol derivatives, increasing metabolite abundance was observed in both cut and non-cut lettuce throughout the storage period. Specifically, significant accumulation of monolignol derivatives was observed in cut and non-cut lettuce after 3 days of storage (Fig. 9C).

In general, we observed that the changes in the metabolite abundances in both cut and non-cut lettuce followed the same trend in response to storage time. This observation suggests that a higher degree of cutting did not trigger different metabolic pathways but appeared to accelerate the reactions. Additionally, the same metabolite response to cutting and storage were observed for Experiment 1 (Supplementary Data, Figs. S2–S3) and Experiment 2, suggesting that the metabolic pathways remain consistent regardless of growing and harvesting periods, ensuring the robustness of our interpretation.

## 4 Discussion

### 4.1 Wound-induced enzymatic browning is progressing during early storage in MAP

Injuries such as cutting trigger a cascade of wound responses, including the formation of signaling molecules such as lysophospholipids, oxylipins, and jasmonates (Laxalt and Munnik, 2002). Jasmonate is a plant hormone derived from C18 fatty acids, which regulates plant growth and metabolism in response to various stress factors including wounding, light, and drought (Wasternack and Song, 2017). In lettuce, similar to other plant species, jasmonate has been reported to activate the expression of the enzyme phenylalanine ammonia lyase (PAL), leading to the accumulation of phenolic metabolites (Jacobo-Velázquez et al., 2015; Kim et al., 2007). Phenolic metabolites including caffeic acid derivatives, flavonoids, and monolignol derivatives are known to be major contributors to post-harvest quality deterioration in fresh-cut lettuce (Mai and Glomb, 2013; Saltveit, 2018b). These secondary metabolites play crucial roles in plant defense mechanisms and are involved in various physiological processes. Their biosynthesis occurs via the phenylpropanoid pathway, which is initiated by conversion of phenylalanine to cinnamic acid, catalyzed by the enzyme PAL.

**Fig. 10:**
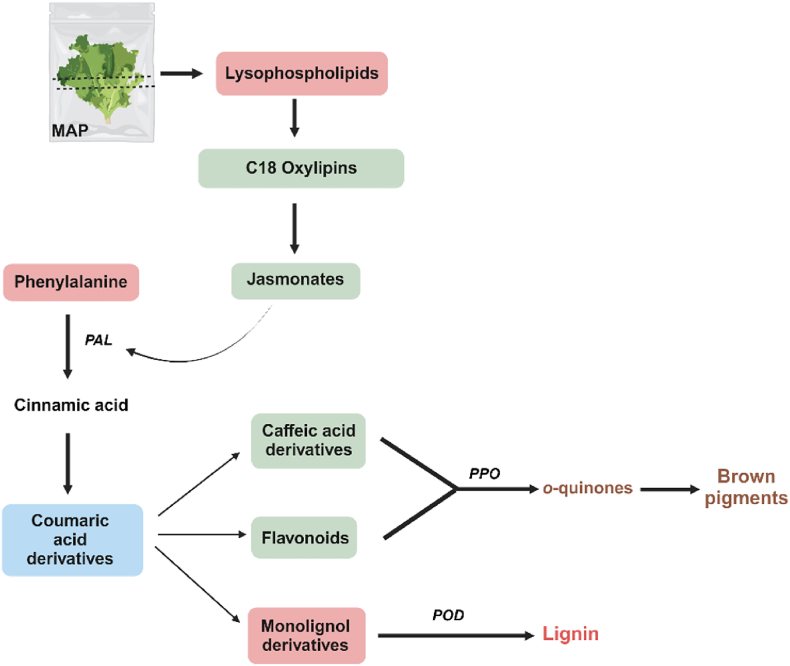
Phenylpropanoid pathway illustrating the metabolites changes in cut and non-cut lettuce stored in MAP. Different color boxes indicate metabolite abundances trends over a 22-day storage period. Red = increasing trend; blue = decreasing trend; green = increasing trend during early storage period (≤3 days), followed by decreasing trend. PAL = Phenylalanine Ammonia Lyase, PPO = Polyphenol Oxidase, POD = Peroxidase.

In this study, we identified the discriminant metabolites differentiating the lettuce samples extracts upon cutting and storage in MAP. The observed changes in these identified metabolites were consistent with the phenylpropanoid pathway, as illustrated in Fig. 10. Here, we propose that cutting released lysophospholipids from cell walls which were then hydrolyzed and oxidized into oxylipins. In non-cut lettuce, these compounds were released during initial tissue wounding when the leaves were detached from the lettuce head. However, in cut lettuce, a greater accumulation of lysophospholipids and oxylipins were detected especially right after cutting, most likely because there were substantially more wounding sites (Fig. 8A). In line with the accumulation of oxylipins, the formation of jasmonates quickly followed, which also occurred in a higher degree in cut leaves (Fig. 8A). Their accumulation peaked after 3 h or 1 day of storage for both cut and non-cut lettuce (Fig. 8B) after which a rapid decrease was observed. Accumulation of jasmonates was likely followed by PAL activation, initiating the phenylpropanoid pathway.

The first indication of an active phenylpropanoid pathway was the decrease in the abundance of coumaric acid derivatives throughout storage (Fig. 8B), which aligns with their role as reactive reaction intermediates during transformation of phenylalanine to caffeic acid derivatives, flavonoids, and monolignol derivatives (Fig. 10). Furthermore, a faster decrease in coumaric acid derivatives was observed in cut lettuce compared to non-cut lettuce (Fig. 8A), suggesting that this pathway is accelerated by the degree of wounding.

In accordance with the latter, activation of the phenylpropanoid pathway following wounding was also indicated by accumulation of caffeic acid derivatives and flavonoids in cut lettuce (Fig. 9A and B), which resulted in the significant progression of browning particularly within the first 3 days of storage (Fig. 3). Caffeic acid derivatives and flavonoids, particularly those with *o*-diphenols group, are the metabolites that are associated with enzymatic browning (García et al., 2017; Liu et al., 2021; Saltveit, 2018b). The enzyme PPO oxidizes these compounds into *o*-quinones and further polymerizes them into compounds forming brown pigment. These compounds act as a barrier against further injury and damage to the plant tissue and promote wound healing. These observations suggest that despite the low levels of oxygen in MAP, enzymatic browning remains the preferred pathway in response to cutting particularly in the early storage.

### 4.2 A shift towards lignification is observed when enzymatic browning is inhibited

In response to storage time, the greatest accumulation of caffeic acid derivatives and flavonoids was observed in cut lettuce samples stored for 3 days but no significant changes in the metabolite abundances were noted after that (Fig. 9A and B). This suggest that enzymatic browning was suppressed after 3 days of MAP storage which was also apparent based on visual observation and measurement of degree of browning of the samples (Fig. 2, Fig. 3). Inhibition of enzymatic browning could be attributed to limited oxygen content in MAP, suppressing the activity of PPO, as reported in a previous study (Luna et al., 2016).

Interestingly, the inhibition of enzymatic browning coincided with significant accumulation of monolignol derivatives observed in both cut and non-cut lettuce. Monolignol derivatives are not substrates for PPO and thus do not contribute to enzymatic browning. Instead, monolignol derivatives are the main building blocks for lignification process. This observation suggests a shift in the phenylpropanoid pathway towards lignification after 3 days of storage. Following this shift, lignification became the prevalent pathway thereafter and continuous accumulation of monolignol derivatives was observed until the end of 22-day storage period (Fig. 9C).

The shift to lignification following inhibition of enzymatic browning is particularly intriguing. A previous study reported on a negative correlation between caffeoylquinic acid and sinapaldehyde derivatives, suggesting a competitive relationship between the two pathways (García et al., 2019). Therefore, it is likely that under the conditions of our study, inhibition of enzymatic browning might induce the shift towards lignification to some extent. In accordance with the latter, given that the biosynthesis of caffeic acid derivatives, flavonoids, and monolignol derivatives all originate from the same phenylpropanoid pathway, it is plausible that when enzymatic browning is suppressed, the pathway is redirected towards monolignol biosynthesis (Fig. 10).

Indeed, lignification has been implicated in the wound healing process. In response to stress, lignification promotes wound healing by providing structural support, sealing of the wounded area, and facilitating tissue repair to prevent further injury and pathogen infection (Barceló, 1997; Bhuiyan et al., 2009). In line with our findings, accumulation of monolignol derivatives has been reported in cut romaine lettuce stored up to 5 days under normal atmosphere (García et al., 2017, 2019). Lignification has also been reported in wounded iceberg lettuce tissue after 7 days storage in association with the occurrence of ethylene-induced russet spotting (Ke and Saltveit, 1988, 1989).

Furthermore, along with monolignol derivatives, we also observed accumulation of lysophospholipids in both cut and non-cut lettuce, particularly during extended storage (after 3 days) (Fig. 8B). This provides evidence that tissue deterioration or changes to cell wall integrity were occurring in late storage, which likely signal a wound response leading to lignification. Therefore, a shift towards lignification could be crucial for sustaining the wound healing process as storage progresses.

Nevertheless, it remains unclear why lignification only became significant during the later stages of storage, while other wound responses, enzymatic browning, occurred early in storage. This implies that lignification is either a systemic response to wounding, even in the less wounded non-cut lettuce, or that it results from a second activation of the phenylpropanoid pathway due to other deterioration events, such as senescence. The preference toward enzymatic browning during early storage could also imply that within this period, enzymatic browning occurs much faster than lignification despite the low level of oxygen in MAP, making it the preferred pathway for quicker wound healing. Further studies are warrantied to investigate the role of MAP in the bipartite response to wounding and extended storage as well as to identify the signaling mechanisms that drive the lignin biosynthesis.

### 4.3 Lignification may alter visual, texture, and flavor qualities in lettuce

In relation to visual quality, lignification can potentially result in pinking of lettuce. A recent study identified two distinct and negatively correlated discoloration phenotypes, pinking and browning, occurring during post-harvest deterioration of lettuce (Hunter et al., 2022). While browning was correlated with accumulation of caffeic acid derivatives, as discussed above, development of pinking was found to be correlated with an increase in transcription of caffeic acid o-methyltransferase, a key enzyme involved in the biosynthesis of monolignols. Although we observed accumulation of monolignol derivatives throughout storage, it was not possible to visually observe development of pinking and objectively differentiate between pinking and browning with the used methodology. Nevertheless, this hypothesis warrants future investigation.

Lignification can also cause alteration in cell wall tissues which potentially lead to texture and flavor changes in lettuce. Previously, lignification has been reported to alter the texture and flavor of fruits and vegetables, negatively affect their edible qualities. For example, lignin accumulation led to hardening of snow peas (Li et al., 2022) and Chinese flowering cabbage stems (Wang et al., 2020) during post-harvest storage. Additionally, lignin accumulation was shown to negatively affect the flavor of asparagus (*Asparagus officinalis* L.) (Schäfer et al., 2016). Understanding the correlation of lignification with texture and flavor changes in lettuce and the extent to which this affects consumer acceptance is of great interest and warrants further investigation through sensory analysis and consumer studies.

From this study alone, it remains unclear how MAP storage under limited oxygen affects the lignification process in lettuce. However, limited oxygen inhibits the activity of peroxidase (POD), the enzyme that catalyzes the lignification process (Tomás-Barberán and Espín, 2001; Yang et al., 2022). Therefore, it is likely that lignification occurs at a slower rate in MAP. This indicates that storage in MAP would not only improve the visual quality of lettuce by inhibiting enzymatic browning, but it could also improve its edible quality by limiting lignification. This hypothesis warrants further investigation.

## 5 Conclusion

Using non-targeted LC-MS metabolomics, we evaluated the changes in metabolite profiles of lettuce upon cutting and extended storage in MAP. A bipartite response to wounding was observed. In early storage, enzymatic browning was the main response upon wounding. correlated well with hyperspectral imaging and spectrophotometric analysis results. After 3 days of MAP storage, enzymatic browning was inhibited and a shift to lignification became apparent and continued to progress throughout the 22-day storage period.

This study provides deeper insights of lettuce deterioration mechanism in MAP storage and its implication toward visual qualities. The results from this study give beneficial information to improve vegetable processing and packaging strategies to prolong the shelf life of fresh-cut vegetables.

## CRediT authorship contribution statement

**Fanny Widjaja:** Conceptualization, Data curation, Formal analysis, Investigation, Methodology, Writing – original draft. **Priscille Steensma:** Conceptualization, Investigation, Methodology, Writing – review & editing. **Leevi Annala:** Conceptualization, Data curation, Formal analysis, Methodology, Software, Writing – review & editing. **Arto Klami:** Conceptualization, Writing – review & editing. **Saijaliisa Kangasjärvi:** Conceptualization, Supervision, Writing – review & editing. **Mari Lehtonen:** Conceptualization, Supervision, Writing – review & editing. **Kirsi S. Mikkonen:** Conceptualization, Funding acquisition, Supervision, Writing – review & editing.

## Declaration of generative AI and AI-assisted technologies in the writing process

During the preparation of this work the authors used ChatGPT in order to improve readability. After using this tool/service, the authors reviewed and edited the content as needed and take full responsibility for the content of the published article.

## Funding

This study is funded by 10.13039/501100009708Novo Nordisk Foundation (Project Number: NNF21OC0066760).

## Declaration of competing interest

The authors declare the following financial interests/personal relationships which may be considered as potential competing interests: Fanny Widjaja reports financial support was provided by 10.13039/501100009708Novo Nordisk Foundation. If there are other authors, they declare that they have no known competing financial interests or personal relationships that could have appeared to influence the work reported in this paper.

## Data Availability

Data will be made available on request.
