# Supplementary material for: Non-targeted LC-MS metabolomics reveal shifts from wound-induced enzymatic browning to lignification during extended storage of fresh-cut lettuce in modified atmosphere packaging
Source: Curr Res Food Sci. 2024 Dec 15;10:100959. doi: 10.1016/j.crfs.2024.100959 (PMC11721850; doi:10.1016/j.crfs.2024.100959)
Supplement: Multimedia component 1 [file mmc1.docx]

**Appendix A. Supplementary Data**

**Non-targeted LC-MS metabolomics reveal shift from wound-induced enzymatic browning to lignification during extended storage of fresh-cut lettuce in modified atmosphere packaging**

Fanny Widjaja^1*^, Priscille Steensma^1^, Leevi Annala,^1^ Arto Klami,^2^ Saijaliisa Kangasjärvi,^3,4,5^ Mari Lehtonen^1^, and Kirsi S. Mikkonen^1,6^

1. Department of Food and Nutrition, University of Helsinki, P.O. Box 66, 00014, Helsinki, Finland.
2. Department of Computer Science, University of Helsinki, P.O. Box 68, 00014, Helsinki, Finland.
3. Organismal and Evolutionary Biology Research Programme, Faculty of Biological and Environmental Sciences, University of Helsinki, P.O. Box 65, 00014, Helsinki, Finland.
4. Department of Agricultural Sciences, Faculty of Agriculture and Forestry, University of Helsinki, P.O. Box 65, 00014, Helsinki, Finland.
5. Viikki Plant Science Center, University of Helsinki, P.O. Box 65, 00014, Helsinki, Finland.
6. Helsinki Institute of Sustainability Science (HELSUS), University of Helsinki, P.O. Box 65, 00014, Helsinki, Finland.

*Corresponding author: fanny.widjaja@helsinki.fi

**Table S1. MS/MS fragments of identified metabolites and their relative abundances**

| # | Tentative ID | Calculated m/z  [M-H]^-^ | | MS/MS fragments observed  (% relative abundance) | |
| --- | --- | --- | --- | --- | --- |
| *Lysophospholipids* | |  | |  | |
| 1 | PE(18:3/0:0)(1) | 474.263 | | 277.218 (100), 255.231 (11), 152.993 (5) | |
| 2 | PE(18:3/0:0)(2) | 474.263 | | 277.218 (100), 255.231 (35),152.993 (32) | |
| 3 | PE(18:2/0:0)(1) | 476.278 | | 279.229 (100), 277.218 (7), 152.993 (22) | |
| 4 | PE(18:2/0:0)(2) | 476.278 | | 279.229 (100), 277.218 (15), 152.993 (12) | |
| 5 | PE(16:0/0:0) | 452.278 | | 255.231 (100), 152,993 (21) | |
| *Oxylipins: C14 and C16 oxylipins* | |  | |  | |
| 6 | Hydroxytetradecanedioic acid | 273.171 | | 155.069 (100), 255.162 (56), 237.150 (47), 111.081 (35) | |
| 7 | Hydroxyhexadecanedioic acid | 301.202 | | 183.100 (100), 201.111 (53) | |
| *Oxylipins: C18 oxylipins* | |  | |  | |
| 8 | Trihydroxyoctadecadienoic acid | 327.218 | | 211.130 (100), 229.141 (64), 171.100 (18), 183.136 (15) | |
| 9 | Trihydroxyoctadecenoic acid | 329.233 | | 211.130 (100), 229.141 (57), 183.136 (13) | |
| *Jasmonates* | |  | |  | |
| 10 | 12-Hydroxy-9,10-dihydrojasmonic acid | 227.129 | | 209.117 (100), 182.953 (72), 136.945 (49), | |
| 11 | Jasmonoyl isoleucine | 322.202 | | 130.087 (100), 183.012 (12) | |
| *Amino acids and Vitamin B5* | |  | |  | |
| 12 | Phenylalanine | 164.072 | | 147.045 (100), 103.052 (33) | |
| 13 | Tryptophan | 203.083 | | 116.051 (100), 142.067 (50), 159.093 (18) | |
| 14 | Vitamin B5 | 218.103 | | 146.083 (100), 88.040 (66) | |
| *Phenolic metabolites: coumaric acid derivatives* | | |  | |  |
| 15 | Coumaroyltartaric acid | 295.046 | | 163.039 (100), 119.049 (76), 149.006 (19) | |
| 16 | Coumaroylquinic acid (1) | 337.093 | | 191.051 (100), 163.039 (5) | |
| 17 | Coumaroylquinic acid (2) | 337.093 | | 191.051 (100), 163.039 (2) | |
| *Phenolic metabolites: caffeic acid derivatives* | |  | |  | |
| 18 | Caffeoyltartaric acid | 311.041 | | 135.045 (100), 149.006 (78), 179.034 (74) | |
| 19 | Caffeoylquinic acid (1) | 353.088 | | 191.051 (100) | |
| 20 | Caffeoylquinic acid (2) | 353.088 | | 191.051 (100) | |
| 21 | Dicaffeoyltartaric acid | 473.073 | | 179.034 (100), 149.006 (85), 135.045 (45), 311.038 (16) | |
| 22 | Dicaffeoylquinic acid | 515.120 | | 191.057 (100), 353.087 (80), 179.034 (54), 135.045 (36) | |
| *Phenolic metabolites: flavonoids* | |  | |  | |
| 23 | Quercetin-3'-glucoronide | 477.068 | | 301.037 (100) | |
| 24 | Quercetin-3'-(6"-acetylglucoside) | 505.099 | | 300.023 (100), 301.030 (50) | |
| *Phenolic metabolites: monolignol derivatives* | |  | |  | |
| 25 | Sinapoyl glucoside | 385.114 | | 207.031 (100), 222.050 (30) | |
| 26 | Ferulic acid-O-glucoside | 355.104 | | 178.024 (100), 193.048 (96) | |
| 27 | Sinapyl alcohol | 209.082 | | 179.034 (100), 161.021 (54), 194.057 (52), 151.039 (44) | |
| 28 | Sinapaldehyde | 207.066 | | 177.071 (100), 192.042 (78), 149.022 (25), 121.030 (11) | |
| 29 | Coniferin | 341.124 | | 164.044 (100), 179.069 (63) | |
| 30 | Syringaresinol | 417.156 | | 181.050 (100), 166.025 (86), 151.001 (55), 387.107 (53) | |

**Figure S1. PLS-DA score plots (Experiment 1 samples; A: non-acidic extracts, B: acidic extracts) describing the differences in metabolite profiles of lettuce due to cutting and storage under MAP. Cut and non-cut lettuce are denoted as (•) and (**▲**), respectively, different colors refer to different storage times (n = 3).**

**
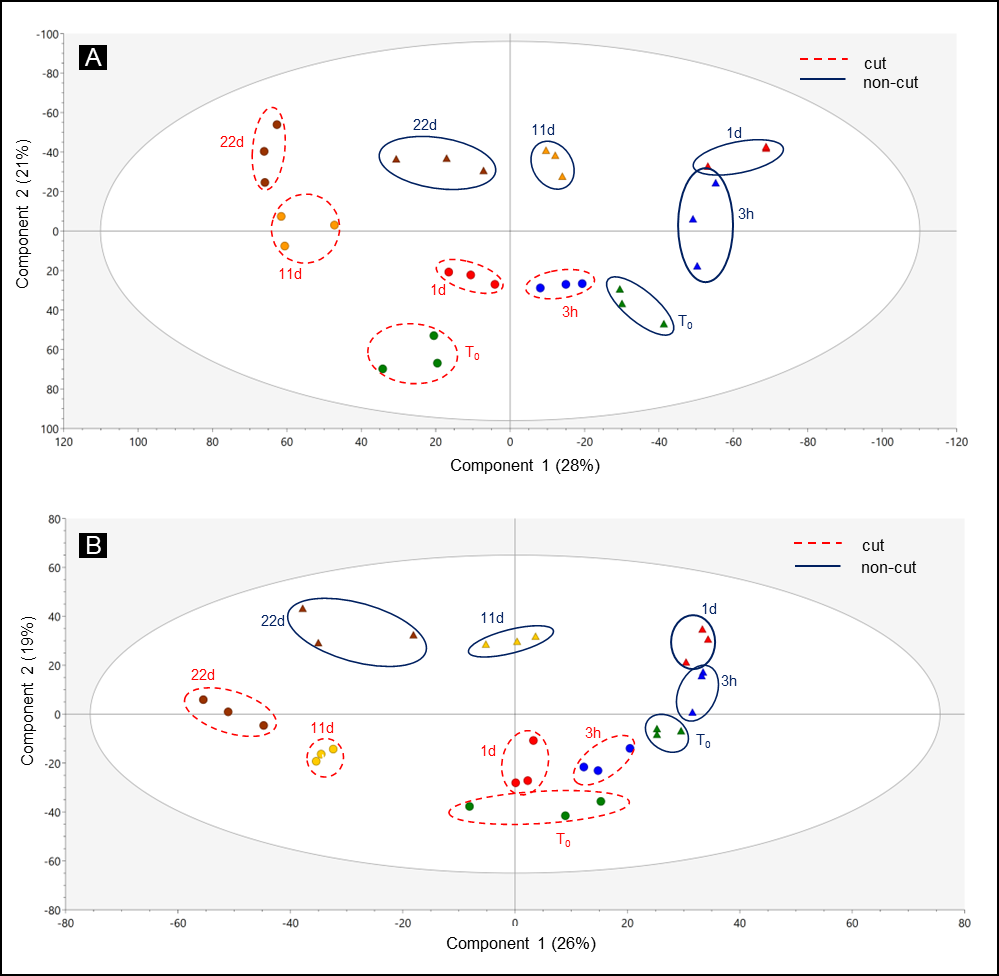
**

**Figure S2. Metabolite response to cutting (A) and storage in MAP (B) from Experiment 1 samples. Heatmaps represent the log_2_ fold change (FC) of metabolite abundances between A) cut vs. non-cut lettuce at each storage time (T_x_) and B) stored (T_x_) vs. fresh (T_0_) for both cut and non-cut lettuce. The data represent the mean of three biological replicates per storage time. Statistical analysis was performed using a 2-tailed Student’s t-test, where * indicates p ≤ 0.05.**


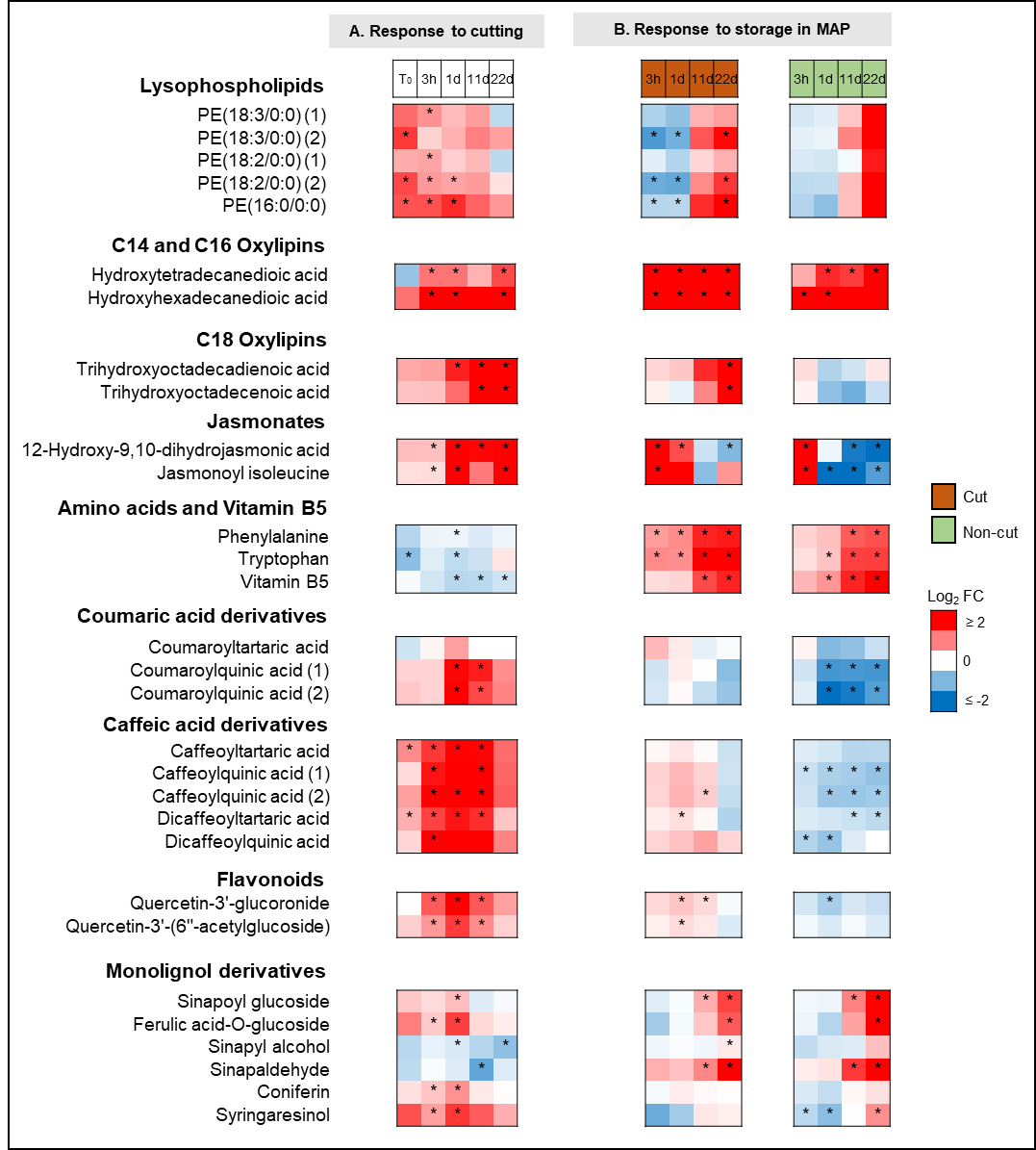


**Figure S3. Accumulation of caffeic acid derivatives (A), flavonoids (B), and monolignol derivatives (C) in cut and non-cut lettuce stored in MAP (Experiment 1). Relative changes are represented in comparison to fresh non-cut lettuce samples (T_0 non-cut_). The data represent the mean ± SD of three biological replicates per storage time. Statistical analysis was performed using a 2-tailed Student’s t-test, where *a* indicates p ≤ 0.05 when compared to non-cut samples and *b* indicates p ≤ 0.05 when compared to T_0_ samples.**

**
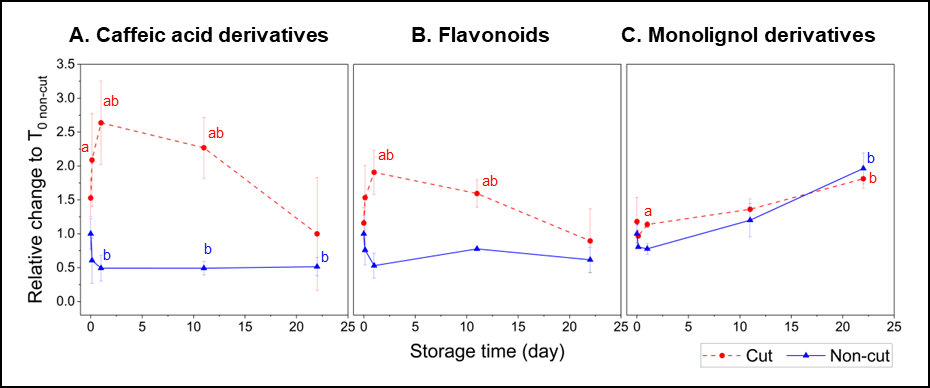
**

**Figure S4. MS/MS spectra of Compound 1 and 2 (Table 1) identified as isomers of PE(18:3/0:0).**

**
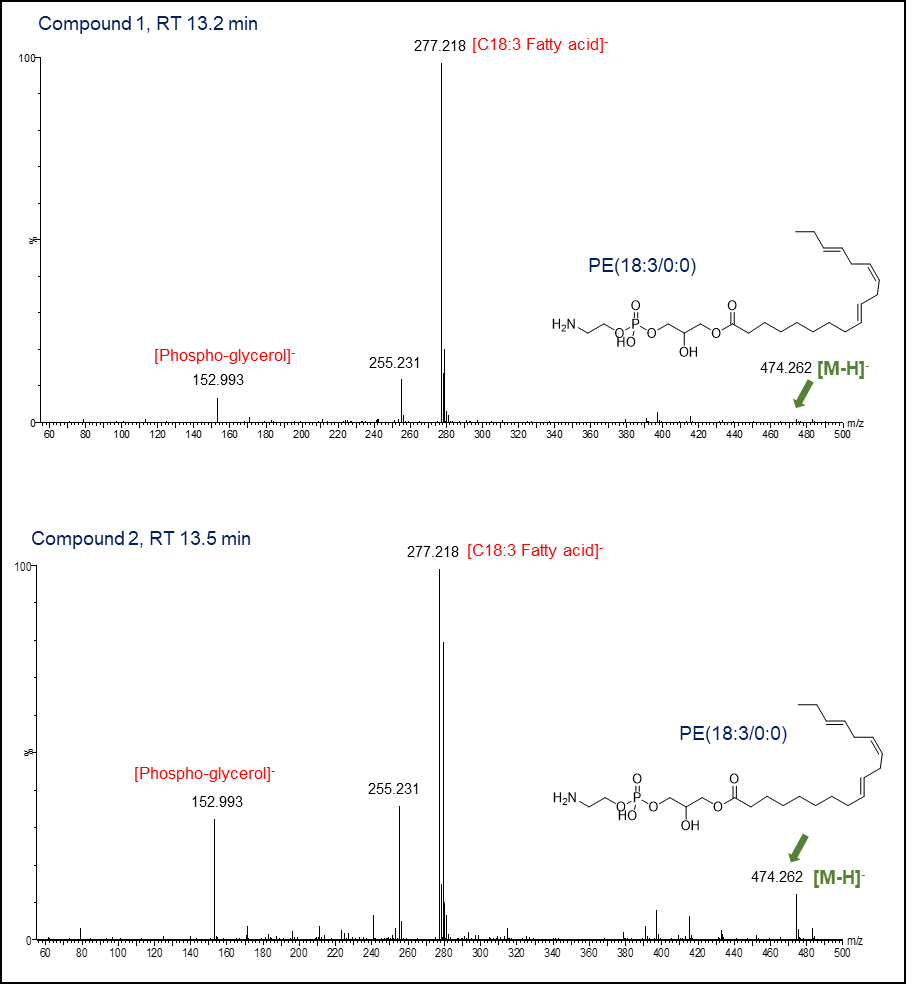
**

**Figure S5. MS/MS spectra of Compound 3 and 4 (Table 1) identified as isomers of PE(18:2/0:0).**

**
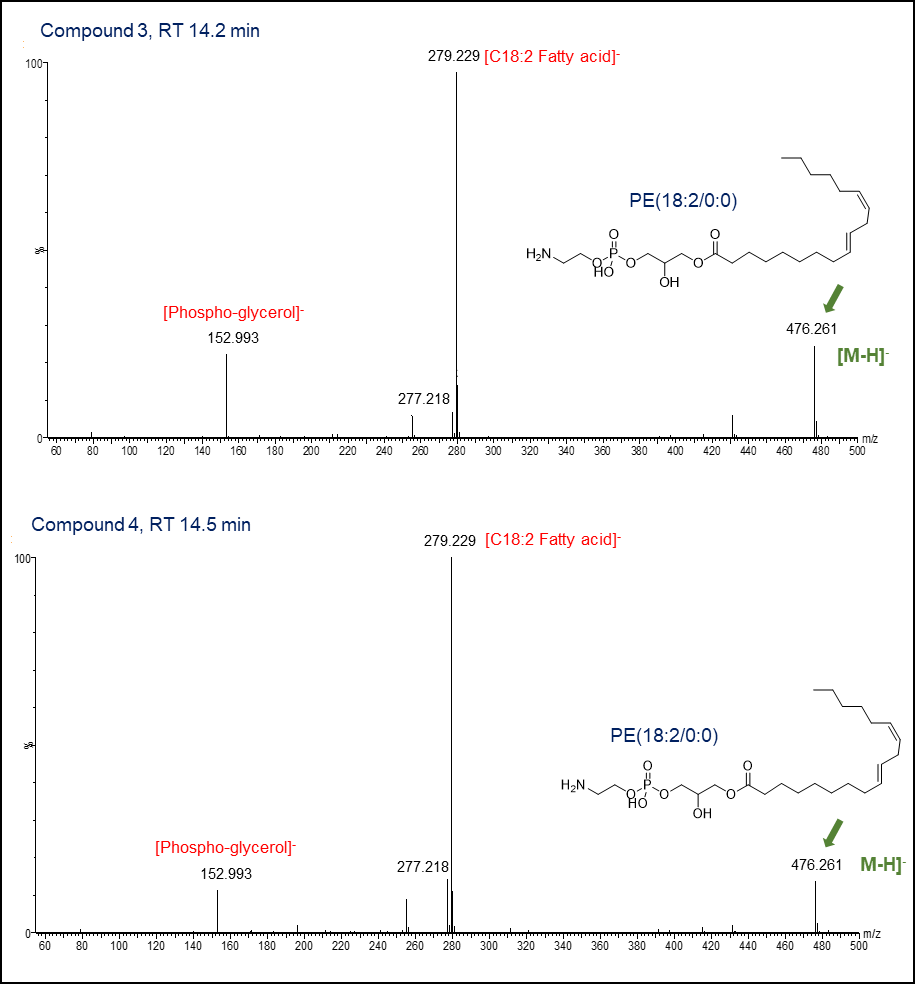
**

**Figure S6. MS/MS spectrum of Compound 5 (Table 1), identified as PE(16:0/0:0).**

**
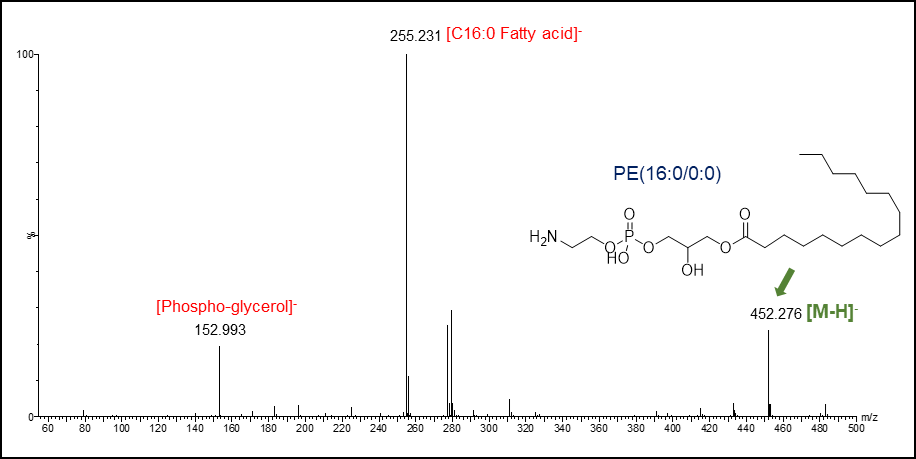
**

**Figure S7. MS/MS spectrum of Compound 6 (Table 1) identified as hydroxytetradecanedioic acid.**

**
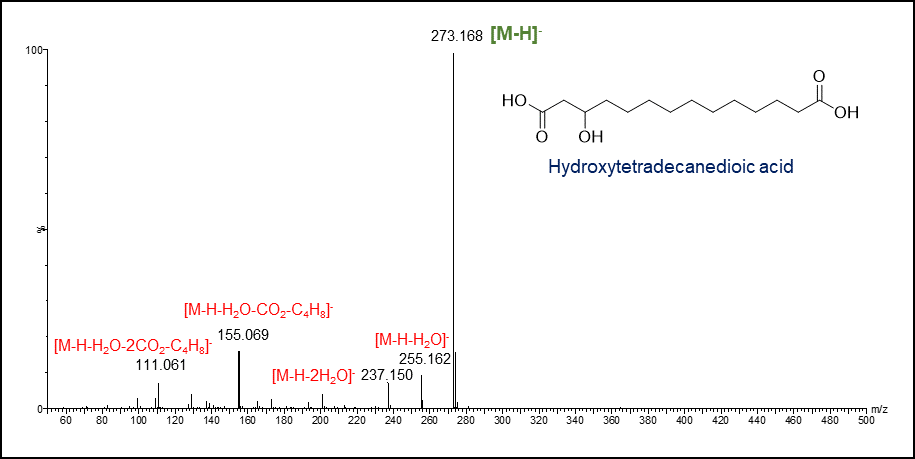
**

**Figure S8. MS/MS spectrum of Compound 7 (Table 1) identified as hydroxyhexadecanedioic acid.**

**
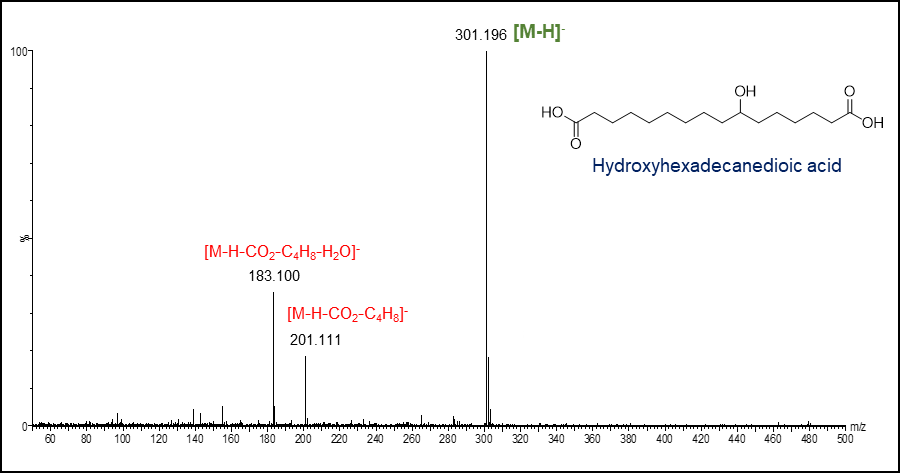
**

**Figure S9. MS/MS spectrum of Compound 8 (Table 1) identified as trihydroxyoctadecadienoic acid.**

**
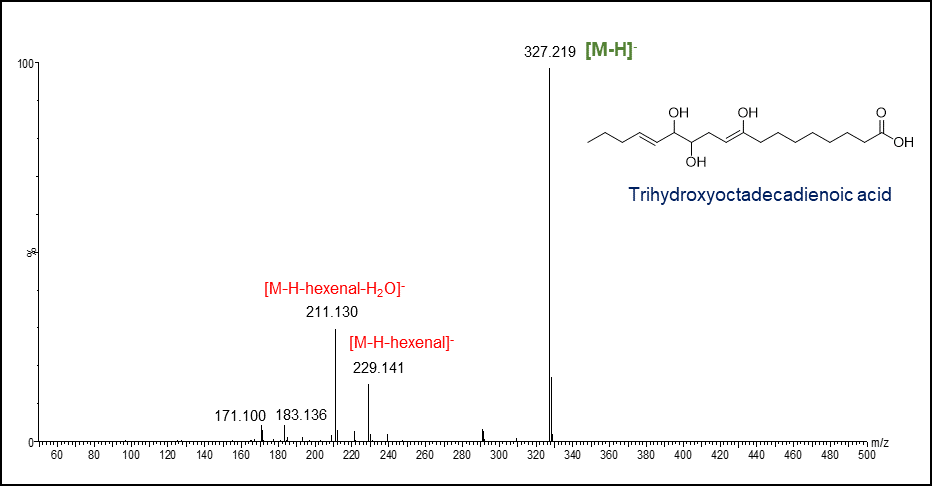
**

**Figure S10. MS/MS spectrum of Compound 9 (Table 1) identified as trihydroxyoctadecenoic acid.**

**
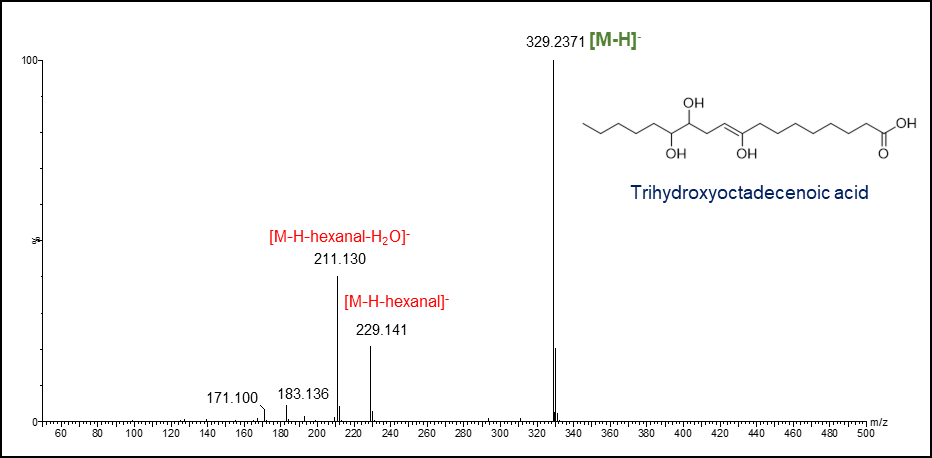
**

**Figure S11. MS/MS spectrum of Compound 11 (Table 1) identified as jasmonoyl isoleucine.**

**
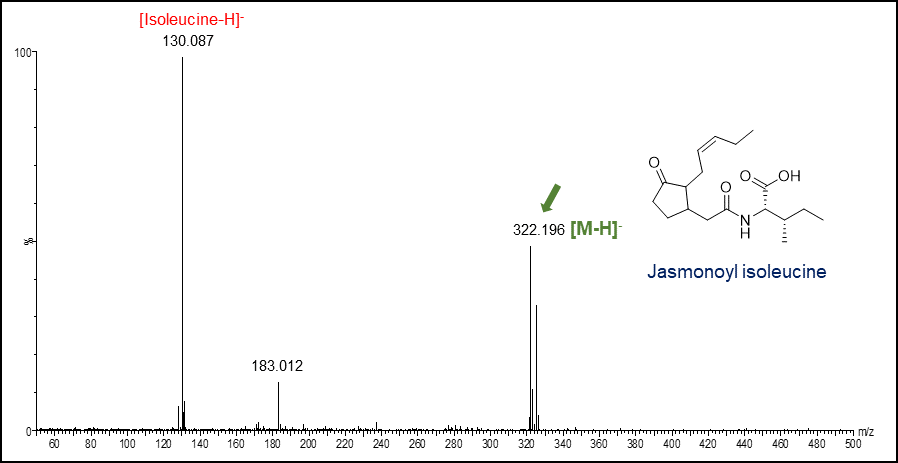
**

**Figure S12. MS/MS spectrum of Compound 15 (Table 1) identified as coumaroyltartaric acid.**

**
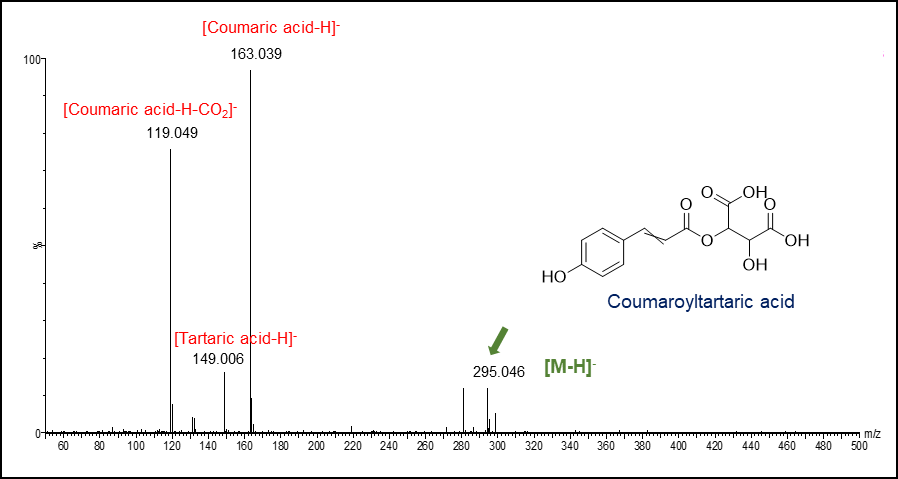
**

**Figure S13. MS/MS spectra of Compound 16 and 17 (Table 1) identified as isomers of coumaroylquinic acid.**

**
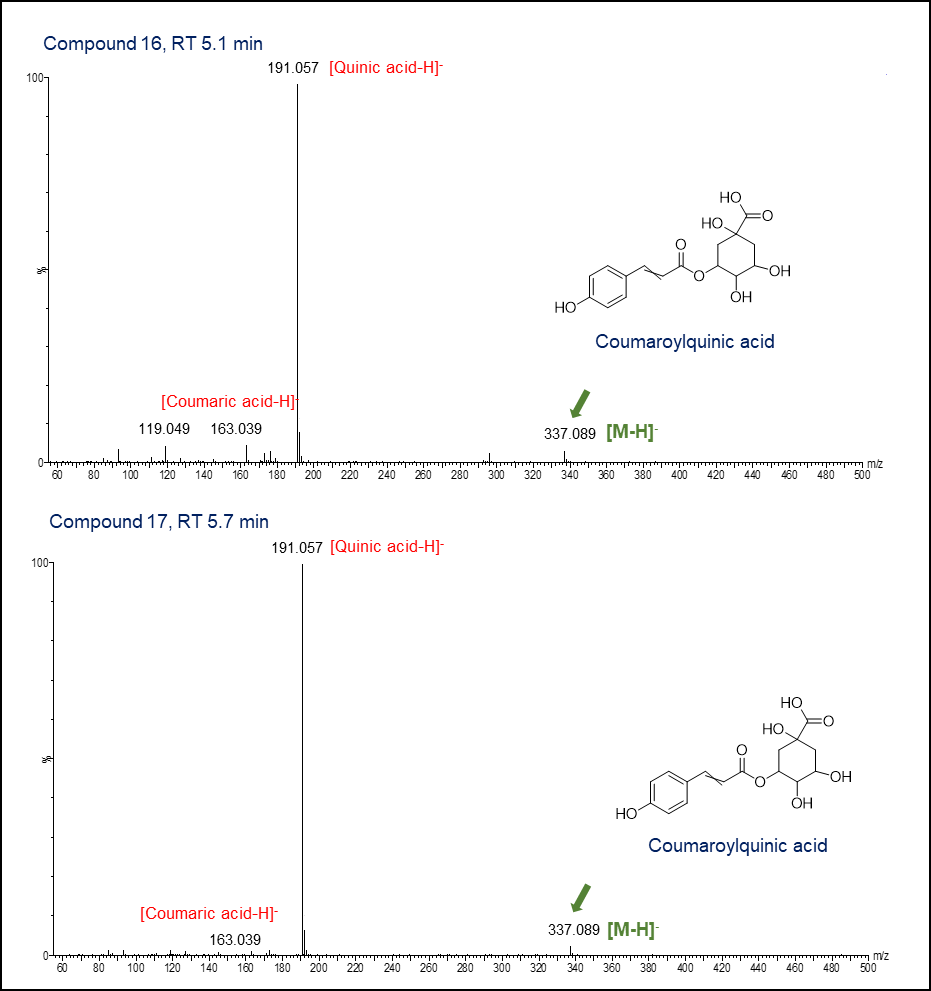
**

**Figure S14. MS/MS spectrum of Compound 18 (Table 1) identified as caffeoyltartaric acid.**

**
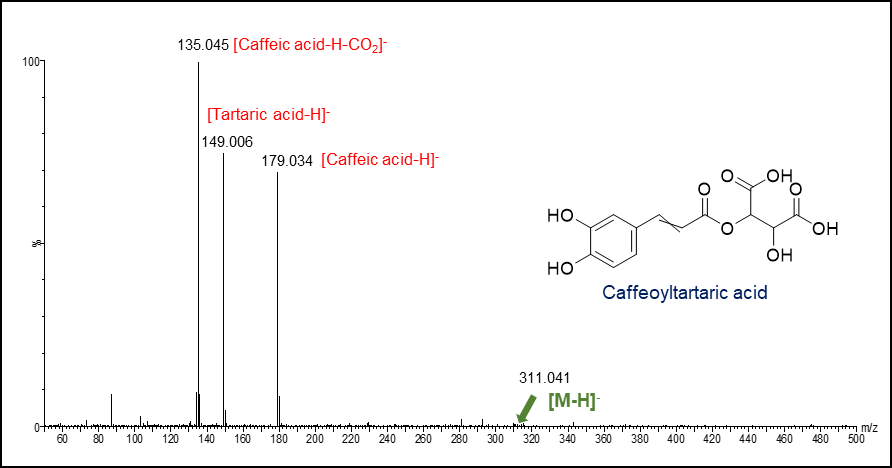
**

**Figure S15. MS/MS spectra of Compound 19 and 20 (Table 1) identified as isomers of caffeoylquinic acid.**

**
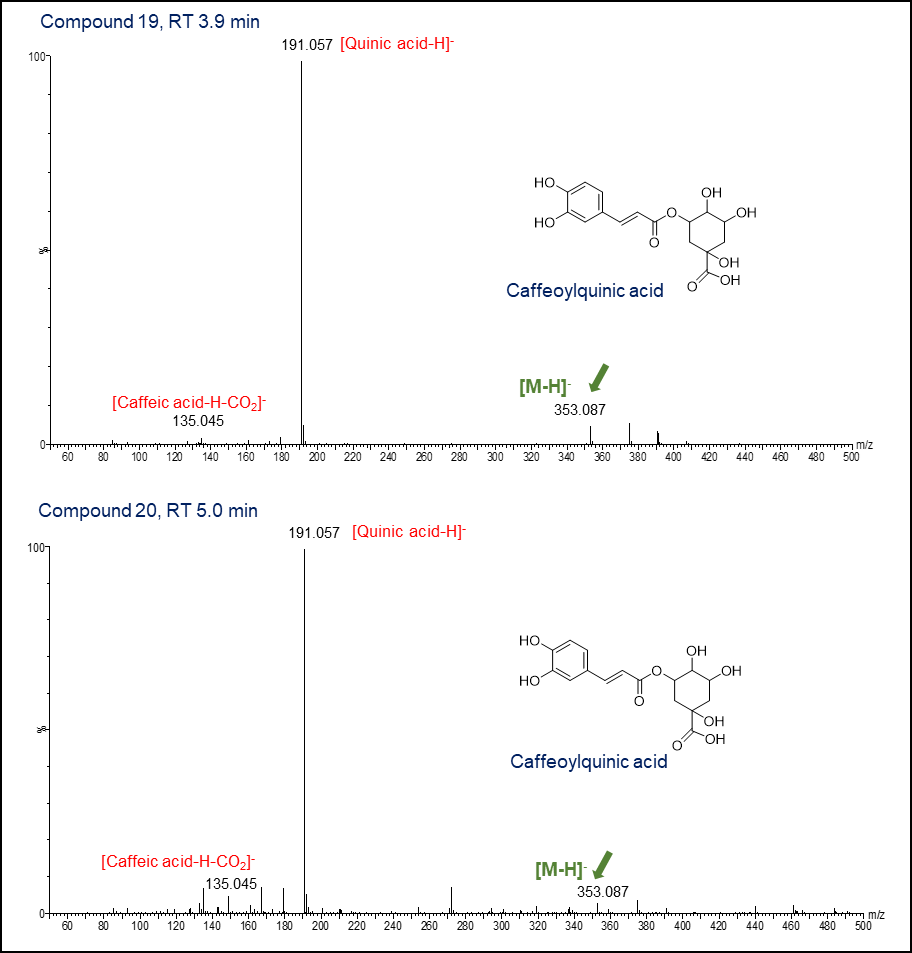
**

**Figure S16. MS/MS spectrum of Compound 21 (Table 1) identified as dicaffeoyltartaric acid.**

**
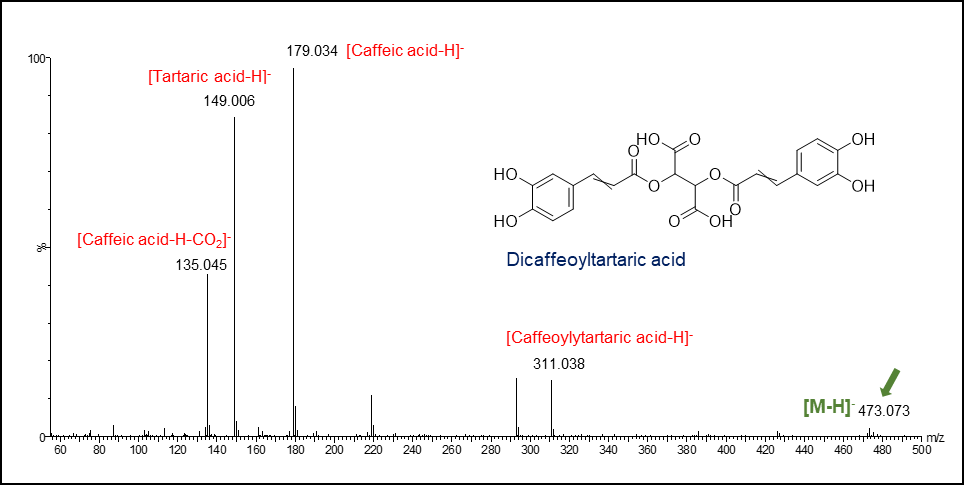
**

**Figure S17. MS/MS spectrum of Compound 22 (Table 1) identified as dicaffeoylquinic acid.**

**
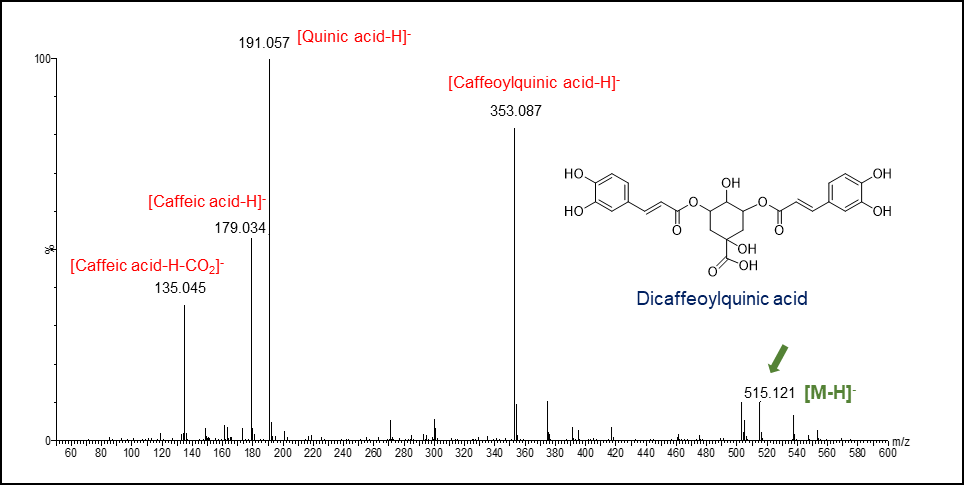
**

**Figure S18. MS/MS spectrum of Compound 23 (Table 1) identified as quercetin-3’-glucuronide.**

**
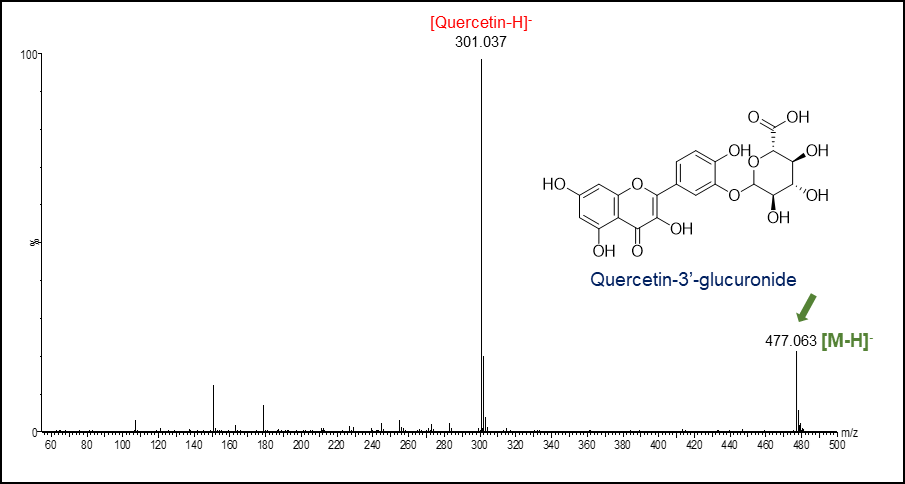
**

**Figure S19. MS/MS spectrum of Compound 24 (Table 1) identified as quercetin-3’-(6”-acetylglucoside).**

**
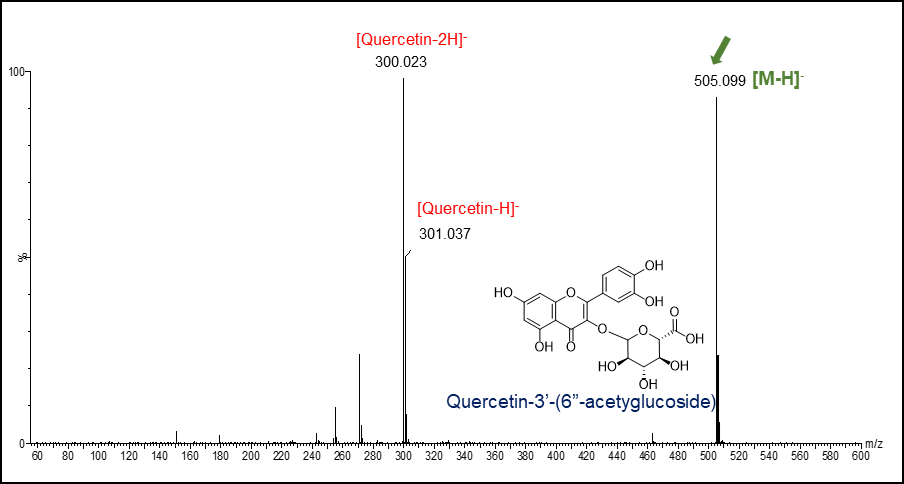
**

**Figure S20. MS/MS spectrum of Compound 25 (Table 1) identified as sinapoyl glucoside.**

**
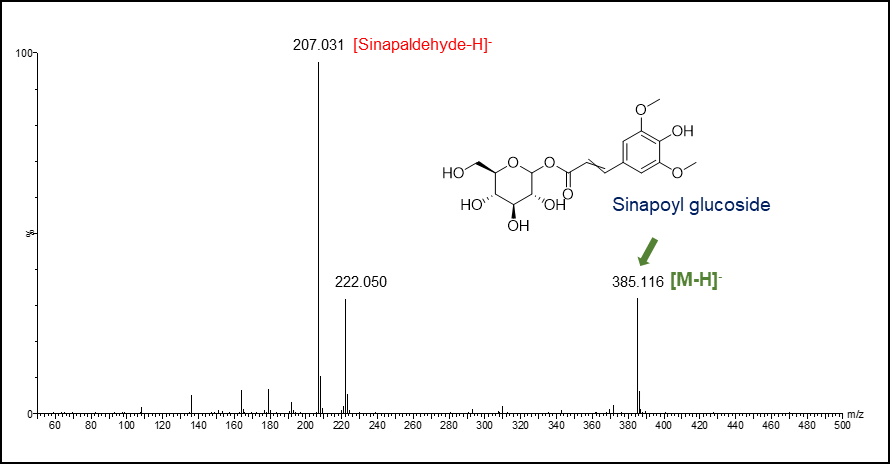
**

**Figure S21. MS/MS spectrum of Compound 26 (Table 1) identified as ferulic acid-O-glucoside.**

**
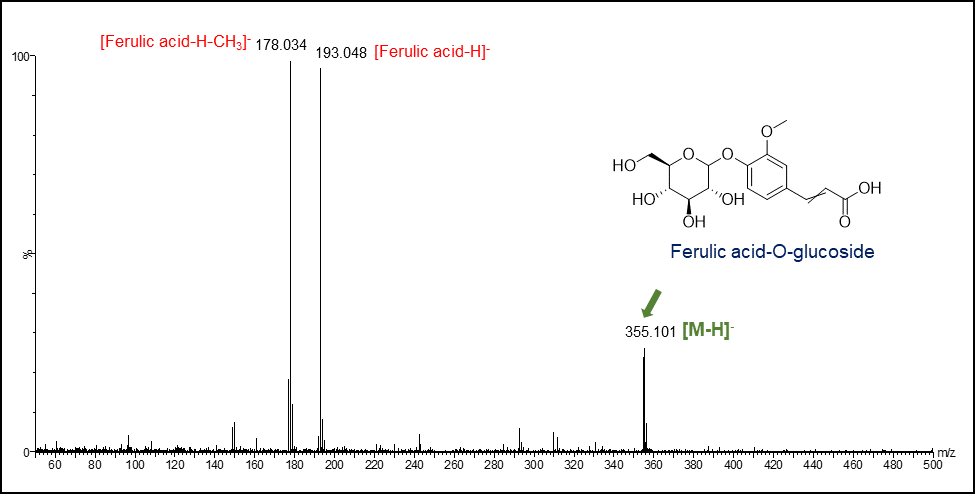
**

**Figure S22. MS/MS spectrum of Compound 28 (Table 1) identified as sinapaldehyde.**

**
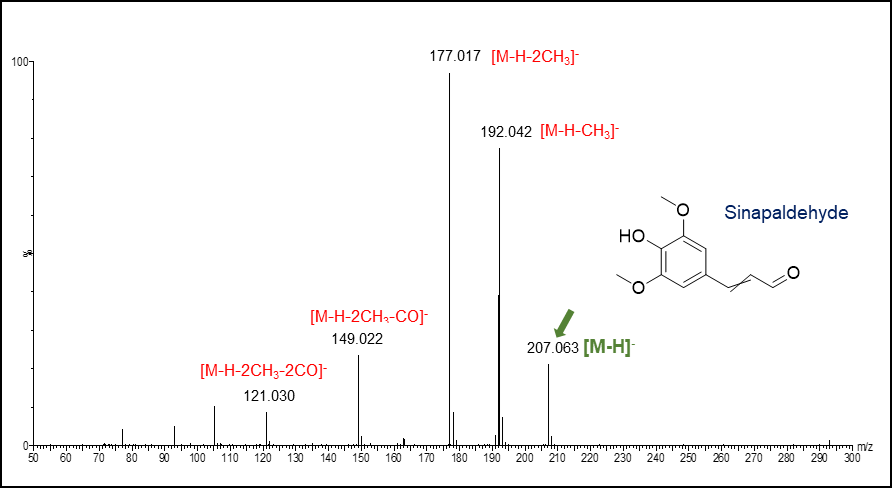
**

**Figure S23. MS/MS spectrum of Compound 29 (Table 1) identified as coniferin.**

**
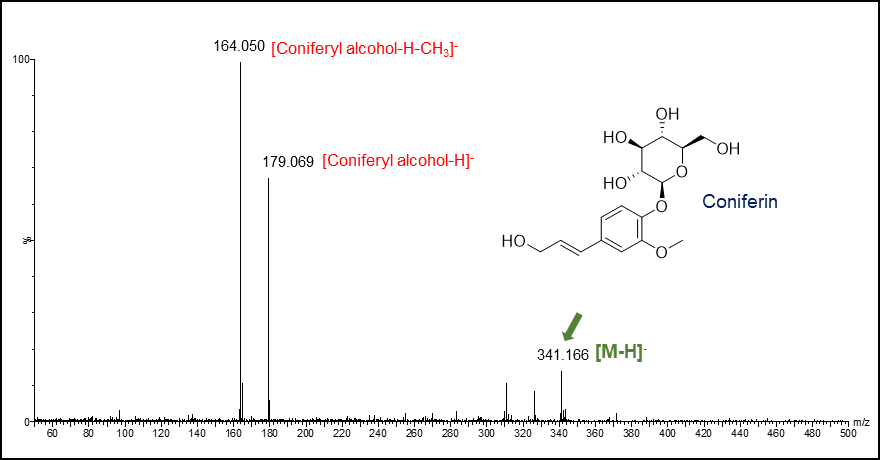
**
